# Evidence-Based Monitoring of *Prodiplosis longifila* in Foliage Crops: Damage, Economic Impact, Trapping Tools, and Population Dynamics

**DOI:** 10.1007/s13744-026-01379-6

**Published:** 2026-04-15

**Authors:** A. Natalia Naranjo-Serrato, William Fernando Cardenas-Urrego, Héctor Julio Villamil-Martha, Luz Mary Pardo-Ramirez, Joaquín Guillermo Ramirez-Gil

**Affiliations:** 1 Laboratorio de Agrocomputación y Análisis Epidemiológico, Departamento de Agronomía, Facultad de Ciencias Agrarias, Universidad Nacional de Colombia, Sede Bogotá, Bogotá, Colombia; 2 Economía Agrícola – Agropecuaria Villapard, Cachipay, Cundinamarca Colombia

**Keywords:** Kernel density, Injury, Color traps, Butcher’s-broom (*Ruscus aculeatus*), Laurel-leaf cocculus (*Cocculus laurifolius*), Monitoring, Spatial-temporal evaluations

## Abstract

*Prodiplosis longifila* (Diptera: Cecidomyiidae) has recently emerged as a major threat in foliage production systems in Colombia; however, key aspects of its damage, population dynamics, and monitoring remain poorly understood. This study addresses these knowledge gaps by evaluating phenological stages, injury patterns, economic impact, and spatio-temporal population dynamics of *P. longifila* in the foliage crops *Ruscus aculeatus* and *Cocculus laurifolius* under open-field and greenhouse conditions in commercial production areas. Monitoring efficiency was assessed using traps of different colors and heights to optimize sampling protocols and improve understanding of adult behavior. Field data were complemented with spatio-temporal analyses to characterize dispersal patterns and aggregation processes within production systems. Damage was detected across all three larval instars, with the most severe injury occurring during the first and second instars, resulting in significant reductions in foliage quality and marketability. Productivity losses reached 53% in *R. aculeatus* and 29% in *C. laurifolius*, accompanied by sharp declines in extra-quality stems and maximum income losses of 79% and 56%, respectively. Adults exhibited a consistent preference for black, white, and yellow traps placed 10-30 cm above host plants, providing practical guidelines for improving monitoring accuracy. Kernel density analyses revealed that populations initially aggregated along greenhouse borders and subsequently dispersed toward the interior, generating internal hotspots associated with localized environmental and microclimatic conditions. Adult activity was predominantly crepuscular, with clear peaks between 21:00-22:00 h and 03:00-04:00 h. This study provides the first integrated assessment of *P. longifila* in foliage crops, generating critical ecological and applied information to support improved monitoring, early detection, and targeted management strategies for this emerging pest in ornamental foliage production systems.

**Supplementary Information:**

The online version contains supplementary material available at 10.1007/s13744-026-01379-6.

## Introduction

The production of ornamental foliage has experienced significant growth worldwide due to the growing demand for these products in the global flower industry (Adebayo et al. 2020). Colombia is a country with an important tradition in the export of flowers and foliage; this sector has been consolidated as a key economic activity for rural development and foreign exchange generation (Patel-Campillo 2010). In the country, the past 15 to 20 years have seen an increase in foliage crops, especially in the municipalities of Cachipay, Zipacón, Anolaima, and La Mesa, in the Cundinamarca region of the Andes mountain range (Valbuena-Gaona et al. 2025). The fronds of these plants are exported, whether directly or through bouquet businesses operated by flower farms in the Sabana de Bogotá. In this region there are about 2,000 ha in production and about 500 small producers, many of them women heads of household (Valbuena-Gaona et al. 2025).

Plants introduced into new production environments typically face few phytosanitary problems during initial establishment; however, as crops expand, pest populations often become established and require management (Velasco et al. 2020). This pattern has been observed in rapidly expanding foliage crops in Cundinamarca, where the unexpected occurrence of arthropods and phytopathogens has generated uncertainty among producers regarding appropriate management options, often leading to risk-averse and nonstrategic control practices (Valbuena-Gaona et al. 2025). These challenges are compounded by limited knowledge of pest biology, ecology, and population dynamics, which restricts the development of effective integrated management strategies and has promoted a heavy reliance on chemical control, increasing production costs and environmental impacts (Han et al. 2022; Jasrotia et al. 2023).

Recently, an insect pest belonging to the genus *Prodiplosis h*as emerged as a significant phytosanitary problem in foliage crops in Cundinamarca, Colombia, causing severe damage to two ornamental species of high commercial relevance in the foliage industry (Valbuena-Gaona et al. 2025). The first affected species is butcher’s broom (*Ruscus aculeatus* L.), a perennial rhizomatous plant of the family Asparagaceae, native to Europe, North Africa, Western Asia, the Caucasus, and the Atlantic island regions (Royal Botanic Gardens, Kew, 2025). This species is widely valued for the production of dark green, glossy, evergreen sprigs, characterized by erect stems bearing spiny phylloclades, highly reduced scaly true leaves, small entomophilous flowers, and conspicuous red berry fruits, attributes that confer high esthetic and market value in ornamental foliage chains (Royal Botanic Gardens, Kew, 2025). Similarly, damage has been documented in laurel-leaf cocculus (*Cocculus laurifolius* (Roxb.) DC.) (Valbuena-Gaona et al. 2025), a perennial species of the family Menispermaceae, order Ranunculales, native to East and Southeast Asia (Royal Botanic Gardens, Kew, 2025). This species is commercially important due to its large, dark green, leathery leaves, which are highly appreciated in the foliage market for their texture, durability, and visual appeal (Royal Botanic Gardens, Kew, 2025) . The occurrence of *Prodiplosis* damage in these species raises concerns not only for foliage production systems but also for its potential expansion and impact on other economically important crops in the region (Valbuena-Gaona et al. 2025) 

Within the genus *Prodiplosis*, *Prodiplosis longifila* (Gagné, 1986) (Diptera: Cecidomyiidae) is recognized as the most economically relevant species, with reported impacts on commercially important crops such as tomato (*Solanum lycopersicum* L.), asparagus (*Asparagus officinalis* L.), and other Solanaceae species (Gagné 1986; Hernandez et al. 2015). Its economic importance lies in the damage it causes to young shoots, inflorescences, and small fruits, which become deformed and significantly reduce yield (Valbuena-Gaona et al. 2025). Currently, this pest is limited to Ecuador, Peru, Colombia, and the USA, but is considered a potential quarantine species for many areas worldwide (EPPO 2025). This pest has been documented causing yield losses reaching up to 100% in tomato (Geraud-Pouey et al. 2022), 16% in potato and asparagus in Peru (Kroschel et al. 2012; Cedano and Cubas 2012), and 25% in other crops in the USA in the 1990 s (Pena et al. 1987). Intensive pesticide use has also led to resistance in field populations (Mujica and Kroschel 2019). Moreover, immature stages cause the greatest damage but are also challenging to manage due to their small size, short life cycle, and protection within host tissues (Quinaloa Gualpa 2022). Given these conditions, quantifying the extent and type of injury is critical for designing effective management strategies and supporting evidence-based decision-making.

In the case of foliage crops, no detailed studies have yet been carried out that integrate population dynamics of *P. longifila* with the description and quantification of damage. This represents a significant knowledge gap since knowing the exact type of damage, the phenological stage responsible, and the potential economic impact forms the basis for establishing sound management criteria. In this regard, a recent study on foliage, under a citizen science approach, reported that farmers in the Cundinamarca region experience yield losses of up to 40% (Valbuena-Gaona et al. 2025). These losses reflect a considerable economic impact, not only due to reductions in product quality but also because of the high cost of control measures (Valbuena-Gaona et al. 2025). Furthermore, current management strategies rely almost exclusively on chemical controls, utilizing active ingredients such as imidacloprid, spinosad, chlorantraniliprole, among others (Valbuena-Gaona et al. 2025). This heavy dependence on pesticides raises significant concerns regarding long-term environmental sustainability, ecological health, and production costs (Valbuena-Gaona et al. 2025). Understanding the magnitude and type of damage, as well as its precise quantification, is therefore essential for developing evidence-based management strategies. In addition, the lack of effective sampling and population quantification tools is a major challenge for growers, hindering more efficient control of the pest.

Population pattern studies are central to understanding pest dynamics, as spatio-temporal analyses help anticipate crop impacts and support the development of integrated management strategies by reflecting the biological and ecological characteristics of pest species (Kim et al. 2007; Park et al. 2024). In *P. longifila*, research in tomato and asparagus has shown highly dynamic populations, with the most severe outbreaks occurring during dry periods associated with favorable temperature, rainfall, and relative humidity conditions (Hernandez et al. 2015; Geraud-Pouey et al. 2022). Adult activity is predominantly nocturnal, which complicates daytime detection and monitoring (Duque et al. 2018). Consequently, tracking population fluctuations through larval counts and damage incidence on host plants, complemented by adult trapping methods (Pena and Duncan 1992; Camborda et al. 2015), has become essential for developing evidence-based management approaches (Binns et al. 2000).

Traps are among the most widely used tools for quantifying pest populations and play a fundamental role in monitoring and controlling insect species in agricultural systems (López et al. 2012). Their effectiveness relies on physical and chemical principles that attract insects, such as color-based chromotropic traps or baits with pheromones and food attractants (Preti et al. 2021). Chromatic traps are particularly common, as many pest species respond to specific wavelengths of light. Yellow traps are the most widely used due to their high efficiency in capturing a broad range of insects, while blue, white, red, and green traps are also employed depending on the target species (Cañedo et al. 2011; Díaz-Silva 2011; Morales Arroyo 2018; Montgomery et al. 2021). Chromatic attraction is linked to insect photoreceptors and their spectral sensitivities, which influence visual perception and movement toward traps (Schnaitmann et al. 2020). Moreover, trap design variables such as size, color, placement height relative to the plant canopy, and spatial distribution within fields strongly affect capture efficiency (Haniotakis 1986; Rodriguez-Saona et al. 2012; Brar et al. 2012). Understanding these factors and optimizing trap density and arrangement are essential for the accurate estimation of pest abundance and assessment of potential damage risks at the field scale (Roubos and Liburd 2010).

The present study was conducted to address critical knowledge gaps regarding the occurrence and pest status of *P. longifila* in foliage crops (*R. aculeatus* and *C. laurifolius*). Currently, information on the injury profile, level of infestation, economic damage, and key biological and behavioral traits of this species remains scarce, limiting the development of reliable monitoring systems and evidence-based management strategies and limiting decision-making in commercial production systems. Accordingly, this study aimed to (i) characterize damage symptoms, infestation levels, and economic losses under open-field and greenhouse conditions; (ii) assess the efficiency of adult monitoring using traps differing in color and vertical placement; (iii) refine detection tools to elucidate adult activity and host-associated behavior in foliage crops; and (iv) analyze spatial-temporal distribution patterns through trapping data and density-based approaches using information collected from commercial farms. We hypothesized that *P. longifila* exhibits differential responses to trap color and height, resulting in distinct spatial-temporal population structures. Collectively, the results provide an entomological framework to improve pest detection, population assessment, and the implementation of more targeted and efficient management strategies for this emerging pest in ornamental foliage production systems.

## Materials and Methods

### Methodological Approach

**Fig. 1:**
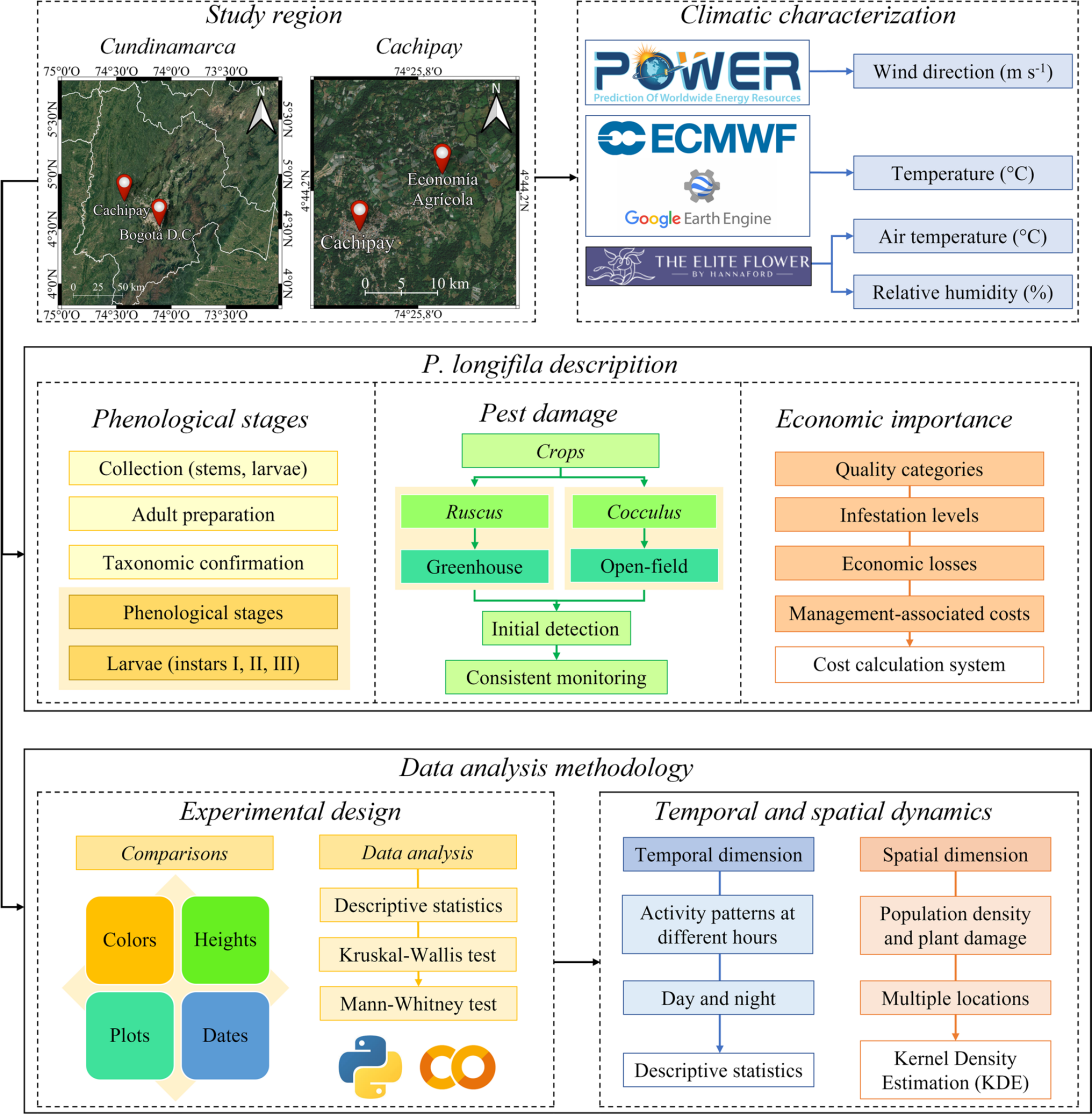
Flowchart of the activities carried out in the study, focusing on climate description of study zone, phenological characterization, and larval damage caused by *Prodiplosis longifila*, evaluation of color traps, and spatio-temporal analysis for evidence-based management of *Ruscus aculeatus* and *Cocculus laurifolius*

Our methodological approach aims to generate baseline information on key ecological, ethological, compartmental, and population-related aspects of *P. longifila* in foliage systems (*R. aculeatus* and *C. laurifolius*), as a foundation for evidence-based decision-making. This approach is particularly relevant given the limited available information on this emerging pest, which has a high potential economic impact on the production systems evaluated (Fig. 1).

In the first stage (Fig. 1), the study focuses on the phenological characterization of the insect at the life stages most relevant for population and damage monitoring, namely larvae, pupae, and adults. This effort seeks to improve recognition and identification, considering the challenges associated with the insect’s small size and the difficulty of accurately distinguishing developmental stages. Subsequently, a basic description of larval damage in *R. aculeatus* and *C. laurifolius* is conducted to support the understanding of its potential effects on yield and product quality, allowing for an initial approximation of the economic impact of this pest in both crops.

In the next stage (Fig. 1), and given the limited knowledge regarding alternative methods for monitoring adult *P. longifila*, the evaluation of different trap types is proposed. Trap characteristics such as color, height, size, and spatial arrangement are assessed to optimize adult monitoring efficiency. Finally, a series of spatial and temporal evaluations of adult population dynamics and associated damage is conducted, integrating analyses across sampling dates and diel (day-night) cycles (Fig. 1). These results provide the basis for understanding pest behavior and for proposing management strategies grounded in the spatio-temporal dynamics of the system.

### Study Region and Basic Description of the Climatic Conditions

**Fig. 2:**
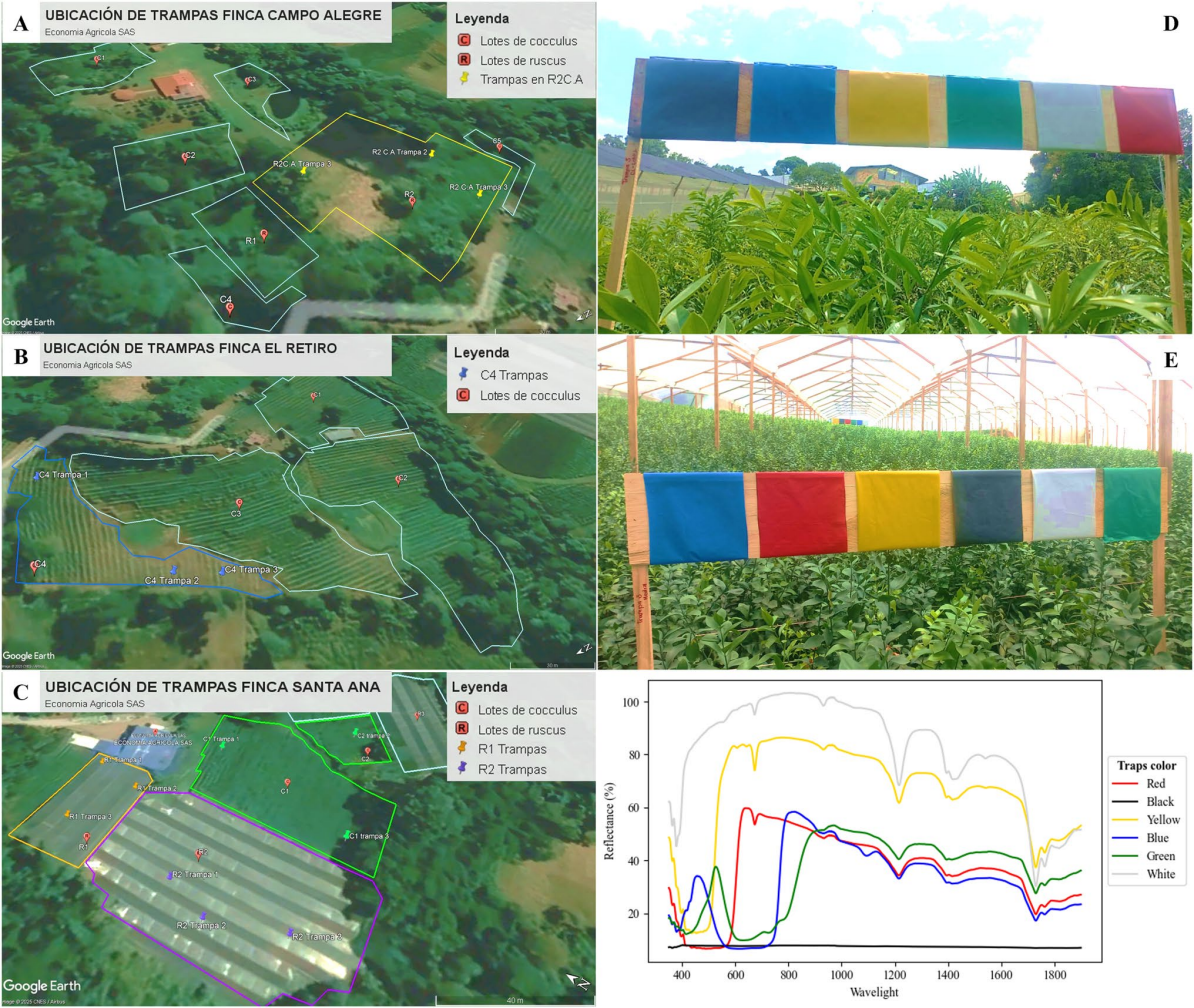
General layout of the plots, trap design, and spectral characterization of the trap colors. **A** Geographical distribution of traps for trial 5 in production plots. **B** Campo Alegre. **C** El Retiro. **C** Santa Ana. **D**–**E** Traps’ structure and colors in *Ruscus aculeatus* in greenhouse, and *Cocculus laurifolius* in open field. **F** Reflectance values of the color traps

Field trials were conducted on farms of the company *Economía Agrícola* in Cachipay, Cundinamarca, Colombia (4°44′17.13″N, 74°25′37.68″W), in both open-field and greenhouse foliage crops of *R. aculeatus* and *C. laurifolius* (Fig. 2). Data from multiple trials were used to evaluate trap color and height based on their efficiency in capturing adults of *P. longifila*. Pest identification was based on morphological and life cycle analyses developed by technical staff from the company *Economia Agricola* (described in detail in later sections), validated by the Museo Entomológico-UNAB at the Universidad Nacional de Colombia in Bogotá, and Instituto Colombiano Agropecuario-ICA inspections. Expert consultation confirmed the likelihood of *P. longifila* presence, but he cautions that, although the current nomenclature is accepted, this genus is under active taxonomic revision, and its classification is subject to future change (Raymond J. Gagné-Personal communication via email, 2024).

All experiments included in this study were conducted under natural population dynamics of *P. longifila* within commercial production systems managed according to standard agronomic practices. Trials were implemented in *R. aculeatus* and *C. laurifolius* crops under conventional production schemes, where pest populations were subject to ongoing phytosanitary, nutritional, and cultural management routinely applied by the company’s technical staff. Phytosanitary management primarily relied on chemical control and included active ingredients such as imidacloprid, spinosad, abamectin, chlorantraniliprole, chlorpyrifos, isocycloseram, lufenuron, lambda-cyhalothrin, cyromazine, methomyl, thiamethoxam, and acephate. Specifically, in *R. aculeatus*, two foliar insecticide applications per week were carried out throughout the production cycle, complemented by monthly drench applications of biological products (e.g., *Beauveria bassiana*, *Bacillus thuringiensis*, *Metarhizium anisopliae*, *Bacillus popilliae*, *Paecilomyces lilacinus*, and *Saccharomyces cerevisiae*), sometimes combined with chemical insecticides. In *C. laurifolius*, one foliar application per week was performed. Crop management also included fertigation twice per week with nitrogen, calcium, and micronutrients, periodic sanitary pruning, weed control, and manual removal of infested stems to reduce larval density. Annual soil fertilization with granular NPK and hilling were applied in both crops. Consequently, the experiments reflect realistic production conditions where *P. longifila* populations interact with agronomic practices, other pests, and diseases.

Climatic characterization focused on the Cachipay and Zipacón producing municipalities of Cundinamarca. Wind direction data were obtained from NASA POWER, while long-term (1980–2021) temperature trends were analyzed using ERA5 reanalysis (ECMWF) (https://www.ecmwf.int/en/forecasts/dataset/ecmwf-reanalysis-v5) accessed via Google Earth Engine (Supplementary Material: Fig. S1). Additional microclimatic data (air temperature and relative humidity (RH)) were recorded every minute in a greenhouse in Cachipay between 2020–2022 by *Elite Flower* company and homogenized to hourly means.

### Description of the Insect’s Phenological Stages, Pest Damage, and Economic Importance of *P. Longifila* in Foliage in the Cundinamarca Area

#### Description of Phenological Stages of the Insect Relevant for Monitoring and Management

The aim of the experiment is not to represent the insect’s life cycle but rather to improve our understanding of the biology of this pest, specifically in the stages that affect foliage, and to provide a basis for improving the taxonomic description of this pest.

Specimens of *P. longifila* for phenological description were obtained from field-collected, naturally infested stems of two *R. aculeatus*. Stems harboring larvae at undetermined instars were carefully selected and transported to the experimental greenhouse. Individual infested stem sections, each containing one or more larvae, were isolated in transparent, vented 1-L plastic containers with sterile sand as substrate and parts of leaves and stems of *R. aculeatus* for food. The base of each stem was placed on a water-saturated floral foam block to maintain turgor, while a separate, moistened filter paper strip within the container provided a microenvironment to prevent desiccation of detached larvae or pupae. Containers were maintained under semi-controlled conditions in a greenhouse. Ambient temperature and relative humidity (RH) inside the greenhouse were recorded daily using a calibrated data logger (HOBO MX2301). Temperature was regulated through passive ventilation and manual relocation of containers to cooler zones within the facility if recorded temperatures exceeded 28°C. No supplemental lighting was provided, following the natural photoperiod of the region.

All containers were inspected daily at 24-h intervals until adult emergence or specimen death. In total, 50 larvae across different larval instars were observed, from which 25 adults were obtained. For each individual, the following data were recorded: (i) developmental stage (larval instar, prepupa, pupa, adult), (ii) stage-specific duration (in days), and (iii) notable morphological changes (e.g., changes in larval cephalic capsule width and body sclerotization, pupal tegument coloration, adult sclerite and wing development). In addition, high-resolution documentation was performed at critical transitional points (e.g., between larval instars, pupation, and eclosion). For general observation and measurement, a digital stereoscopic microscope (Leica S9i, Leica Microsystems) with an integrated 10-megapixel camera and 10:1 zoom ratio was used, typically at 10–40× magnification. For detailed examination of micro-morphological structures (e.g., chaetotaxy, spiracle detail, genitalia), a phase-contrast compound microscope (Nikon Eclipse 50i, Nikon Instruments) equipped with a DS-Fi3 digital camera was employed at 50–400× magnification. All photomicrographs included a calibrated scale bar. Image processing for extended depth of field (focus stacking) and minimal contrast/brightness adjustments was conducted using Helicon Focus 8.0 (Helicon Soft Ltd.) and GIMP 2.10 (GNU Image Manipulation Program), respectively.

The egg stage was not characterized due to its absence in field-collected samples. Larval instars (I-III) were determined based on morphological descriptions from Peña et al. (1989). For taxonomic confirmation and slide preparation, larval and adult specimens were dissected with a ventral incision using a minute pin. Specimens were subsequently cleared in warm 30% KOH, neutralized in 10% acetic acid, dehydrated in a graded ethanol series (70%, 95%), and cleared in clove oil. Cleared specimens were mounted in Canada balsam on glass slides. For adult specimens, heads were mounted for frontal view, and male genitalia were excised for dorsoventral examination. Slides were cured on a hot plate at 50 °C and examined under phase-contrast microscopy. Species identification was performed using the keys of Gagné (1994, 2018). Developmental stage duration is reported as mean ± standard deviation (range). Morphological measurements were obtained from digital images using the measurement modules of Leica Application Suite X or NIS-Elements D software.

#### Description of Damage Associated With Larvae On Foliage

The effects of larvae on different states of plant tissues were evaluated to characterize the lesions and damage caused by *P. longifila* on the hosts *R. aculeatus* and *C. laurifolius*. Monitoring was conducted under uncontrolled field conditions, in which climatic factors corresponded to those that naturally develop within greenhouses or in outdoor environments. Fertilization regimes were consistent with those normally applied in the crop and consisted of fertigation with magnesium sulfate, nitrogen, and micronutrients such as boron, copper, iron, manganese, molybdenum, and zinc in the case of *R. aculeatus*. In addition, cultural practices such as weed control and sanitary pruning, primarily focused on disease management, were implemented, all of which were carried out with technical support provided by *Economía Agrícola* (no details are given regarding products, dosages, or application methods, due to a confidentiality agreement with the company that allowed the research to be conducted).

Study plots were established in commercial beds previously identified, through farm-wide monitoring, as persistent infestation foci. Within these plots, newly sprouting stems exhibiting initial symptoms (e.g., larval presence, early tissue malformation) were tagged and monitored individually. Each tagged stem constituted an observational unit, tracked from initial symptom detection through to the commercial harvest point determined empirically by stem color and texture. Damage was quantified and described through sequential photographic documentation and direct observation. Key descriptors included (i) the precise anatomical location of damage on the stem (e.g., apical meristem, axillary bud, internode), (ii) the phenological timing of symptom appearance relative to stem development, and (iii) the specific morphological alterations induced in the host plant tissue (e.g., gall formation, stem twisting, chlorosis, necrosis). Monitoring was maintained on the same individual stem from larval detection to harvest to ensure temporal consistency and accurately document the complete damage sequence for each host species.

#### Quantification of the Economic Impact Associated With Damage Caused By Larvae to Foliage

An economic impact assessment was conducted, which provides a comprehensive estimate of the total economic burden associated with *P. longifila* infestations, defined as the proportion of stems infested with *P. longifila* individuals relative to the total number of stems harvested at the end of the cycle. This latter datum was established by the company based on production calculations per batch for each quality category (types A, B, and C, also known as Nacional) under conditions without the presence of the pest (Supplementary Material: Table S1). In this assessment, only losses directly attributable to *P. longifila* were considered; damage or quality reductions associated with other phytosanitary problems, biotic or abiotic stressors, or esthetic defects unrelated to *P. longifila* infestation were explicitly excluded from the analysis. The assessment considered both losses associated with reductions in the productivity and quality of harvested stems, as well as additional management costs incurred.

To determine the economic impact of *P. longifila*, a thorough evaluation was carried out in affected greenhouses (Fig. 2). The first step was to define the criteria that determine the commercial quality of stems, which govern their market classification and price. Category A stems are characterized by a mature green coloration and foliage lengths or, in the case of *R. aculeatus*, phyllode length, ranging between 30 and 35 cm (with 35 cm being optimal) (Supplementary Material: Table S1). The apical region must present erect, densely distributed leaves/phyllode. In *C. laurifolius*, foliar discontinuity, also referred to as “windows” in the foliage, is not accepted, whereas in *Ruscus* acceptable deviation from the central stem axis must not exceed 2 cm, and apical leaf burn must not surpass 1 mm; otherwise, postharvest esthetic conditioning is required. Category B stems also exhibit a mature green hue, although in *Cocculus* a slightly lime-green shade is tolerated. The minimum length required is 40 cm, with leaves/phyllode erect or somewhat flattened. Twisting under 3 cm is permissible, as are minor malformations, provided these are not apical. Apically trimmed stems are also accepted if the stem diameter at the cutting point does not exceed 4 mm. Category C stems, commonly known as “Nacional,” display a darker green coloration and may present chlorotic mottling or textural alterations of the foliage, such as stippling caused by *P. longifila* feeding injury (described in detail in the “Results” section). Their minimum length is 45 cm, and in *R. aculeatus*, inflorescences may even be present. Tip burns of up to 3 mm are accepted, as are thin stems with torsion or apical malformations, including apical trimming. In this category, no minimum foliage/frond length is specified, as commonly defined in the sector.

Once the categories were established, plots with three levels of infestation (based on company monitoring records, data not shown because they are part of the company’s confidential information) were evaluated. In *R. aculeatus*, infestation criteria were defined according to percentage of incidence (% of stems affected), with illustrative average densities, where plots with high infestation showed a historical average damage incidence above 24.1%, averaging 9 larvae per stem and 10 adults captured on average in the yellow adhesive traps (50 × 50 cm) deployed in these plots. Medium infestation ranged between 10.1 and 24% damage incidence, with averages of 3–4 larvae per stem and 6 adults per trap. Low infestation plots showed < 10% damage incidence, averaging 1 larva per stem and 1 adult per trap. In *C. laurifolius*, high infestation was defined as > 10.1% damage incidence, averaging 4 larvae per stem; medium infestation ranged between 6.1 and 10% damage incidence, averaging 2 larvae per stem; and low infestation was < 6% damage incidence, with an average of 1 larva per stem. In this case, the adult stage was not quantified due to the absence of monitoring traps in the evaluated plots. The direct impact of *P. longifila* was defined in terms of its effect on foliage productivity and quality. Productivity was assessed based on the number of stems produced per square meter for a production cycle, corresponding to two months in *R. aculeatus* and three months in *C. laurifolius*. Quality was determined by classifying harvested stems into categories A, B, and C, as previously described.

This information was incorporated into the cost calculation system, which allowed for an estimate of the economic losses derived from reductions in both productivity and quality due to *P. longifila* infestation and subsequent injury, damage, and its respective effect on the loss. In addition, management-associated costs were calculated, considering direct expenditures such as inputs, labor, and the time required for pesticide applications.

### Experimental Design for Capturing Adults of *P. Longifila* in Foliage Crops

Populations of *P. longifila* are inherently dynamic; they will always remain dynamic. This is precisely why our work is highly robust; it accounts for all intrinsic and extrinsic variability. To ensure this, it was included data from many years and numerous experiments. Furthermore, the results were not compared across experiments; each one was evaluated independently. As outlined throughout this section, the experimental design was systematically structured to ensure a clear conceptual linkage among all trials, with each experiment addressing a specific objective that collectively contributes to a comprehensive understanding of adult monitoring conditions and population behavior across contrasting production scenarios.

Five independent field trials were conducted during 2021, 2022, and 2024 at commercial foliage farms of Economía Agrícola to evaluate adult monitoring conditions for *P. longifila*. The trials were designed and implemented by the company’s technical team, with methodological advice and supervision from the research group and the participation of a specialist in insect population behavior. Each trial addressed specific combinations of trap color, installation height, crop phenological stage, and production system, with the objective of identifying optimal conditions for adult detection, population estimation, and spatio-temporal characterization (Table 1; Fig. 2; Supplementary Material Fig. S2).

**Table 1:** Description of the trials performed and its characteristics

Trial	Date	Host	Replicates	Average traps	Color of trap	Heights (cm)*	Assessments
1	June 2021	*Ruscus*	5 greenhouses	4	Yellow, white, black	15	Daily × 5 days
2	September 2021	*Ruscus*	6 greenhouses	6	Yellow, white	50–100	Daily × 13 days
3	December 2021	*Ruscus*	5 greenhouses	3	Yellow, white	30–49, 50–69, 70–89, 90–109, 110–129, 130–149, 150–159	Every 2 days × 12 days (6 assessments)
4	January–February 2022	*Ruscus*	5 greenhouses	7	Yellow, black, red	50–69, 60–80, 70–89, 90–109, 110–129, 130–149, 150–169	13 times × 30 days (2–5-day intervals)
5	September–October 2024	*Ruscus*, *Cocculus*	3 greenhouses, 4 fields	5	White, black, yellow, red, green, blue	20	3 times × 30 days (10-day intervals)

^*^For trial 5, the height is established above the crop canopy, while for others is above the ground surface

The experiments were conducted at three farms Campo Alegre (CA), El Retiro (ER), and Santa Ana (SA) (Fig. 2A-–C) on two host species: *R. aculeatus* grown under greenhouse conditions and *C. laurifolius* grown under open-field conditions (Fig. 2E). Trials 1–4 were performed exclusively in protected production systems of *R. aculeatus*, following standardized commercial greenhouse construction, design, and crop management practices. Greenhouses averaged 100 m in length and 50–100 m in width, contained approximately 20 beds per structure, and maintained a planting density of 9 plants m⁻^2^, with bed lengths ranging from 25 to 40 m. Trial 5 included both greenhouse-grown *R. aculeatus* and open-field *C. laurifolius*.

Monitoring was conducted using chromatic sticky traps. Trap dimensions were 20 × 20 cm in trials 1 and 5, and 10 × 10 cm in Trials 2–4, based on previous internal evaluations indicating these sizes as the most cost-effective for adult capture (data not shown due to confidentiality agreement with the company). All traps were coated with 20W50 adhesive oil and mounted on wooden supports. In all trials, traps were replaced prior to each assessment. To minimize positional bias, traps were randomly distributed within beds, avoiding bed edges, and positioned horizontally in the center of each bed following its orientation. Trap color and vertical placement varied among trials to evaluate adult chromatic preference and vertical flight activity relative to canopy structure and crop phenology (Table 1; Supplementary Material Fig. S2). Trap height was measured from ground level in trials 1–4, whereas in trial 5 traps were positioned relative to the crop canopy, at a fixed distance above the foliage.

Trials 1–4 were conducted sequentially in commercial *R. aculeatus* greenhouses between June 2021 and February 2022, each trial addressing specific aspects of adult monitoring under contrasting crop phenological stages and canopy structures (Table 1; Supplementary Material Fig. S2). Trial 1 focused on evaluating adult responses to trap color at a standardized low height corresponding to the lower canopy stratum, with daily assessments conducted over a short period to capture immediate capture patterns. Building on these results, trial 2 examined adult captures at intermediate vertical strata within the active foliage zone, allowing assessment of population fluctuations over a longer monitoring period. Subsequently, trial 3 expanded the evaluation of vertical distribution by testing multiple height strata under conditions of reduced canopy height and foliage density following harvest, providing insight into adult flight activity in structurally simplified canopies. Finally, trial 4 was conducted in mature crops with dense foliage and fully developed canopies, integrating multiple trap colors and a wide vertical range to characterize adult distribution across the entire canopy profile over an extended sampling period. Operational details, including trap colors, heights, replication, and assessment schedules for each trial, are provided in Table 1.

In trial 5, white, black, yellow, red, green, and blue plastic sheets were evaluated in three *R. aculeatus* greenhouses and four *C. laurifolius* open-field plots. *Ruscus aculeatus* plots were selected at Santa Ana (R1, R2) and Campo Alegre (R3) based on infestation levels (high, medium, and low) defined from monitoring data of the same year (Fig. 2A, C). Traps were installed 20 cm above the crop canopy on plants in the vegetative stage with immature shoots. For *C. laurifolius*, three traps per plot were installed in open-field conditions at Santa Ana (C1–C3) and El Retiro (C4) (Fig. 2B), avoiding the first two beds and maintaining a minimum distance of 2 m from plot edges. Colored plastic sheets were arranged randomly on wooden boards, with three to six replicates per site; although initial color order was randomized, the same sequence was maintained throughout the trial (Fig. 2D-–E). 

To complement the color-based trap characterization, the spectral response of each trap color was quantified. Reflectance spectra were obtained using an SR-1900 spectroradiometer (Spectral Evolution, Inc., USA) with a spectral range of 350–1900 nm (nominal resolution: 3.5 nm for 350–1000 nm; 10 nm for 1000–1900 nm). The instrument was calibrated before measurement using a white polytetrafluoroethylene spectral on panel with 99% reflectance (Spectral Evolution, Inc., USA). Ten replicate spectral measurements were taken for each trap color under controlled ambient conditions (18.5 °C, 55% RH). Samples were illuminated by two 400-W halogen lights positioned at 45° angles and 20 cm from the measurement point. The spectroradiometer’s pistol grip accessory was mounted to maintain a fixed measurement geometry: a 90° angle and a 1.5-cm distance from the sample surface. The resulting raw spectral reflectance curves for each color are presented without preprocessing in Fig. 2F.

In all cases, the assessment consisted in the number (count) of adult trap catches. For adult population counts, both complete insects and recognizable body parts (e.g., antennae, legs, thorax) were considered according to taxonomic criteria. To process each trap, the colored plastic surface was detached from the wooden support and the adhesive was carefully removed without touching the treated area. The count was performed in ascending order (from bottom to top and from right to left) to ensure that the entire covered surface was checked. Afterwards, clean plastic sheets were reinstalled on the boards, maintaining the initial color order. In cases where insects were incomplete or identification was uncertain, counts were verified using image magnification with a portable Digital Microscope FC, RoHS CE, equipped with an integrated digital camera and software compatible with mobile devices, checking for complete insects or ≥ 2 connected body parts (thorax + legs + antennae), while isolated fragments were discarded.

Data analysis in all trials consisted of nonparametric statistical analysis after applying the Shapiro–Wilk test for normality and Levene's test for homoscedasticity, and verifying that the assumptions of normal data distribution were not complied with. Kruskal–Wallis test (McKight and Najab 2010; Chirinos et al. 2020) was performed with the *stats* module from the SciPy library, in order to verify statistical differences in the number of adults captured between the features trap color, assessment dates, heights, host, and plots and their interactions, with the median number of adults as the population estimator. If significant differences were detected based on the Kruskal–Wallis test, multiple comparisons were made with the Mann–Whitney test (Kishore and Jaswal 2022), with the *stats* module from the SciPy library, to compare medians between two independent groups, showing bar plots with the total number of adults captured adding statistical annotations using the *Annotator* class from the *statannotations* library, to create a list of all possible combinations of pairs of features without repetition for statistical comparison. Both analyses were made in Python with the Google Colab environment, to evaluate significant differences in features evaluated for each trial.

### Temporal and Spatial Dynamics of Adult Populations and Damage Associated With *P. Longifila* in Foliage

This phase focused on analyzing the spatio-temporal dynamics of *P. longifila* adults and the associated damage in *R. aculeatus* greenhouses. The objective was to characterize the behavior of this pest species in two dimensions: (i) the temporal dimension, by examining activity patterns across different hours of the day and night and (ii) the spatial dimension, through detailed monitoring to assess fluctuations in population density and plant damage at multiple locations within the greenhouse. This dual approach provided a more comprehensive understanding of the species’ behavior under production conditions. To achieve this, two complementary trials were conducted, each designed to capture the variability in both temporal activity and spatial distribution of *P. longifila*. These experiments made it possible to quantify pest population dynamics with greater precision and to identify critical periods and locations of infestation that may inform more effective monitoring and management strategies.

In the first assay (temporal dynamics) and based on previous reports indicating crepuscular activity in *P. longifila* adults (Duque et al. 2018), captures were conducted using yellow sticky traps (40 × 40 cm) positioned at 50 cm above the ground under greenhouse and outside conditions. Traps were coated with 20W50 adhesive oil and supplemented with a white LED light source (840 lumens, 10 W) to enhance attractiveness. The evaluation was performed over a continuous 14-h period, from 17:00 on July 2 to 7:00 on July 3, 2021, with hourly counts of captured adults. This approach allowed the characterization of fine-scale diel activity patterns, providing insights into fluctuations in adult abundance across nocturnal hours. To further assess population activity, color traps were deployed both inside and outside the greenhouse. Ten traps were systematically distributed inside the structure along a 100 m × 50 m grid, while an additional ten traps were installed outside, each located 10 m from the greenhouse perimeter, under equivalent height and conditions, for comparative monitoring of *P. longifila* population density.

To investigate the spatial distribution of *P. longifila*, a systematic grid sampling design was implemented within the greenhouse, dividing the area into twelve 3 m × 4 m quadrants. At the centroid of each grid, paired sticky traps, one yellow and one white (10 × 10 cm each), were installed at 40 cm above the crop canopy, separated by one meter and oriented against the prevailing wind (Supplementary Material: Fig. S2C). Traps were coated with SAFERTAC® glue and checked daily, with adhesive replenished after each reading to maintain capture efficiency. Concurrently, thirty plants per grid were randomly sampled to quantify larval infestation and stem damage, focusing on the number of shoots affected. This dual monitoring scheme allowed the integration of adult captures and larval damage, providing a robust dataset to elucidate the pest’s spatial aggregation patterns.

The temporal dataset was initially analyzed using descriptive statistics, calculating measures of central tendency and dispersion to summarize adult captures at each hourly interval. Specifically, the arithmetic mean, standard deviation, and standard error of the mean were estimated for each monitoring period. This procedure allowed not only the identification of the central trend of *P. longifila* adult activity but also the quantification of variability across hours, thereby providing a robust foundation for detecting peaks in diel activity patterns. Line plots were generated to visualize temporal fluctuations and to assess potential heteroscedasticity in capture data. Variation sources were illustrated using 95% confidence intervals (CI_95_):

1 $$CI_{95}=\overset-{\mathcal x}\pm z\frac s{\sqrt n}$$

where “$\underline{x}$” is the sample mean, “z” is the confidence level value set at 1.96, “s” is the sample standard deviation, and “n” the sample size.

For the spatial dimension, given the discrete nature of the variables (adult captures and stem damage incidence) and the systematic grid sampling design, a point-pattern analysis approach was employed (Cressie, 1993). Spatial dependence and aggregation were first explored using Moran’s I and Ripley’s K-function, which served to statistically confirm non-randomness in the distribution of captures and damage incidence. Following this exploratory stage, the radial basis interpolation method was implemented, specifically the Kernel Density Estimation (KDE) algorithm (Chen 2017), to generate smoothed probability surfaces of pest distribution and damage within the greenhouse environment.

The KDE was parameterized in Python (v3.10) using the *scipy.stats.gaussian_kde* module for continuous density estimation, combined with the function *sklearn.neighbors.KernelDensity* of scikit-learn library to refine bandwidth selection. The bandwidth, a critical parameter controlling the degree of smoothing, was optimized through cross-validation (fivefold) using the likelihood cross-validation criterion, which minimized overfitting and ensured biologically meaningful spatial clusters. This optimization was executed within the Google Colab environment, leveraging GPU acceleration for matrix operations and ensuring reproducibility through version-controlled notebooks.

To validate the robustness of the kernel density surfaces, sensitivity analyses were performed by comparing outputs under different kernel functions (Gaussian, Epanechnikov, and Tophat). The Gaussian kernel yielded the most biologically consistent aggregation patterns, with high-density foci located near greenhouse edges and entry points, aligning with empirical observations of adult and damage hotspots. Resulting density maps were normalized and statistically compared using Pearson correlation coefficients and Root Mean Square Error (RMSE) between predicted density values and observed counts across grids.

## Results

### Characterization of the Climatic Regime and Its Variability in the Cachipay Study Area

Analysis of the local historical climate record reveals a marked warming trend. Over the past 30 years, the average annual temperature has increased by approximately 1.72 °C (from ~23°C to ~25°C), with a pronounced acceleration of ~ 0.91°C in the last 2–3 years. This recent warming rate notably exceeds the 20th-century historical trend for Colombia’s average temperature. Specifically, maximum and minimum temperatures increased by 1.72 °C and 1.0 °C, respectively, over the 30-year period, indicating an asymmetric warming pattern. In contrast, mean relative humidity has remained relatively stable at the decadal scale. At the intra-annual scale, the region’s climate is influenced by the seasonal migration of the Intertropical Convergence Zone (ITCZ) and the El Niño-Southern Oscillation (ENSO) phenomenon, resulting in a bimodal temperature and a unimodal precipitation pattern typical of much of the Colombian Andes. At the diurnal scale, a predictable pattern of local circulation governs microclimatic conditions. Relative humidity shows greater variability between 07:00 and 17:00 local time. Local wind circulation follows a well-defined valley-mountain-valley pattern: nocturnal and early morning flows originate predominantly from the northeast quadrant (0–90°), while daytime winds are more variable in direction due to upslope thermal flows (see Supplementary Material, Fig. S1).

### Description of the Insect’s Phenological Stages, Pest Damage, and Economic Importance of *P. Longifila* in Foliage in the Cundinamarca Area

#### Description of Phenological Stages of the Insect Relevant for Monitoring and Management

**Fig. 3:**
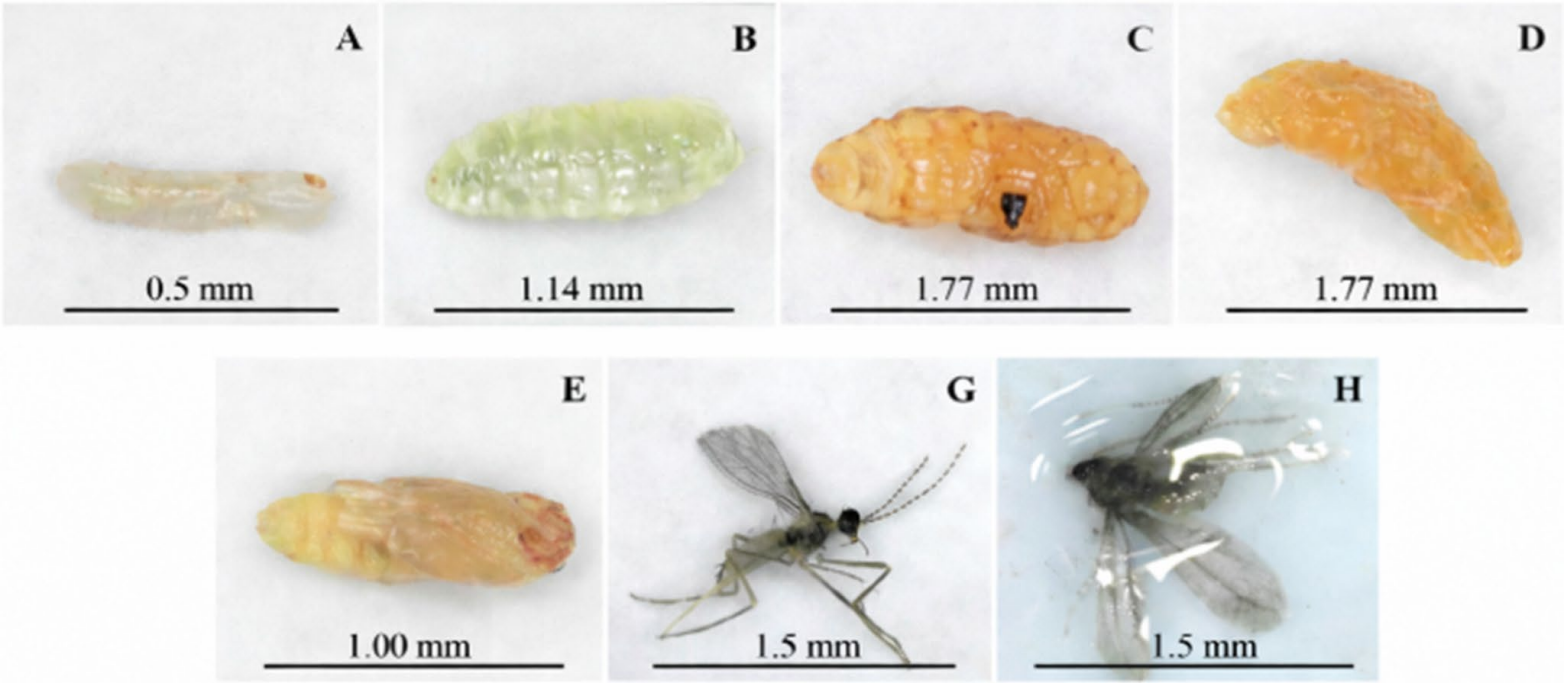
Basic aspects of the morphology of *Prodiplosis longifila* in foliage crops. **A–C** Larval instars I–III. **D** Prepupa. **E** Pupa. **G**–**H** Adults observed under stereomicroscope and field microscope, specimens documented according to a standardized field collection protocol. The egg stage was not observed due to the difficulty of detecting this stage under field conditions. Photographs: *Economía Agrícola*

Under the evaluation of the trials, *P. longifila* comprises three stages: larva, pupa, and adult. The damage associated with *P. longifila* in foliage crops occurs during the larval phase, which goes through three instars (I, II, and III) (Fig. 3). Larva I measures approximately 0.51 ± 0.1 mm in length and has a transparent body (Fig. 3A); larva II reaches an average length of 1.14 ± 0.3 mm and maintains a whitish-transparent coloration (Fig. 3B); larva III reaches 1.77 ± 0.4 mm in length, with a whitish color in the early days, which turns yellow-orange in the final hours before pupation (prepupa) (Fig. 3C and D). These three larval instars last a total of 4 to 7 days, with instars I and II causing the greatest damage to the foliage. Subsequently, the insect enters the pupal stage, lasting 3 to 5 days, where it measures about 1.0 mm ± 0.15 in length (Fig. 3E and 3 F). Finally, the adult reaches approximately 1.5 ± 0.3 mm in length, with antennae composed of 12 flagellomeres and wings with reduced venation and setae (Fig. 3G and H) (for the trials that were conducted, only 4 adults were obtained). Sexual dimorphism is evident in the antennae: males have elongate, binodose flagellomeres with well-developed circumfila, whereas females have shorter, simpler flagellomeres with reduced circumfila. Adult longevity differs between sexes: males live between 2 and 3 days, whereas females live 3 to 5 days.

#### Description of Damage Associated With Larvae On Foliage

**Fig. 4:**
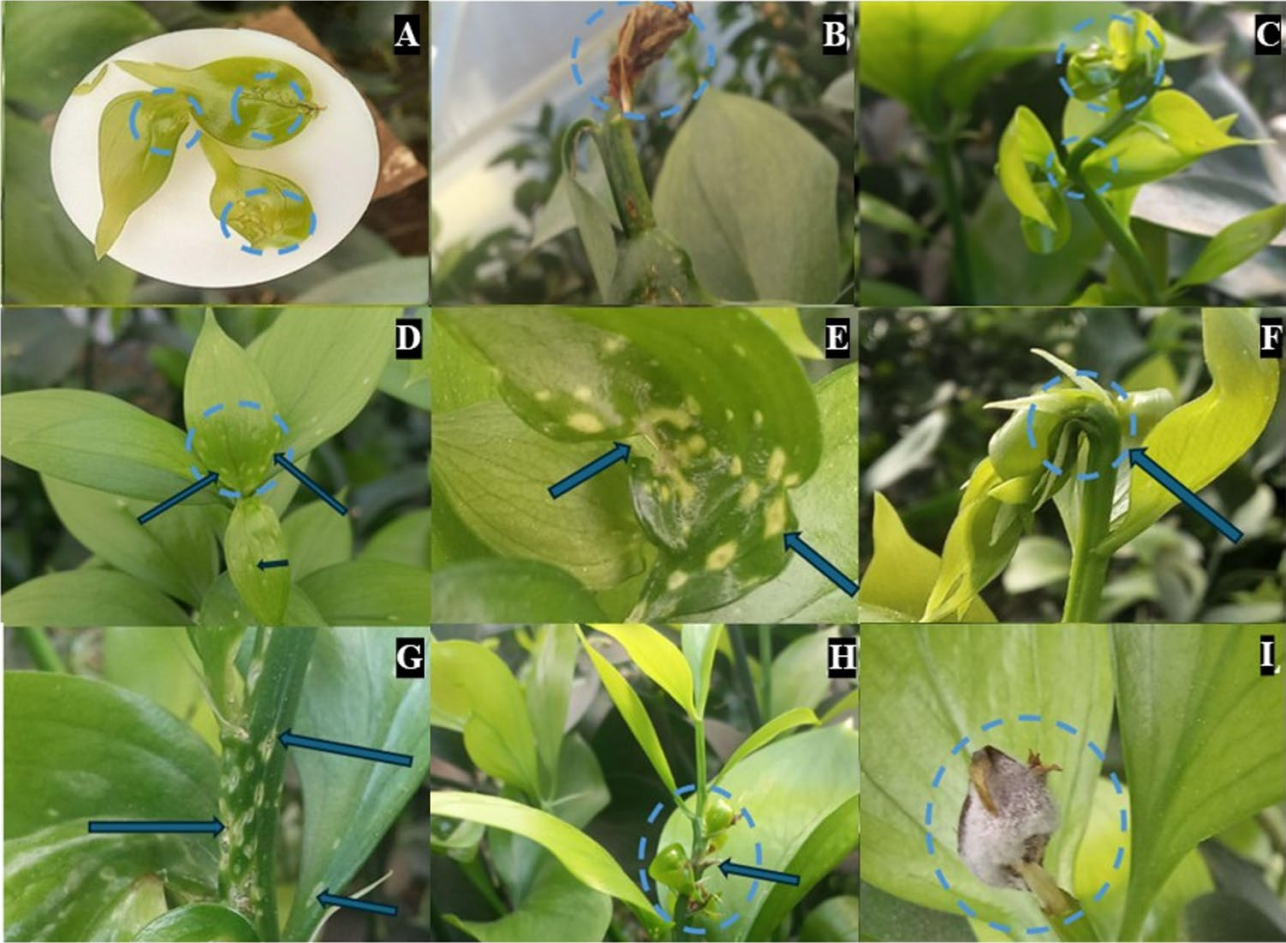
Photographic visualization of the damage caused by *Prodiplosis longifila* in *Ruscus aculeatus*. **A**, **B**, and **C** Shoot apex damage. **D** and **E** Leaf damage. **F** and **G** Stem damage. **H** Severe leaf and stem damage. **I** Fungal colonization associated with *Prodiplosis longifila* damage. Photographs: *Economía Agrícola*

The damage associated with *P. longifila* in *Ruscus* is expressed in different ways depending on where it is located. If damage occurs only at the shoot apex, the leaves are observed with translucent spots (Fig. 4A), are moist to the touch, and later wither and dry up (Fig. 4D), sometimes completely detaching from the stem. This type of damage stops the growth of the breton. However, if damage occurs in leaves below the apex or apical leaves that were affected when they were larger and there was no necrosis, small translucent spots appear that later change to a white color as they mature (Fig. 4D), while if damage was severe, the leaf can become deformed (Fig. 4E). In the case where the larvae generate adherence of the leaf to the stem, as shown in Fig. 4F, and feed on these two zones, a twisted stem with atrophied growth is presented (Fig. 4C) due to damage generated in the vascular bundles. In this case, the leaves usually present translucent spots that later change to a white color, as does the stem (Fig. 4G). In the case of severe damage to both the stem and the leaves, severe deformations occur, accompanied by necrosis in the leaves, which prevents the plant from being useful (Fig. 4H). Finally, *P. longifila*, although not a direct form of damage, allows or gives rise to potential secondary attacks due to fungal diseases (Fig. 4I).

**Fig. 5:**
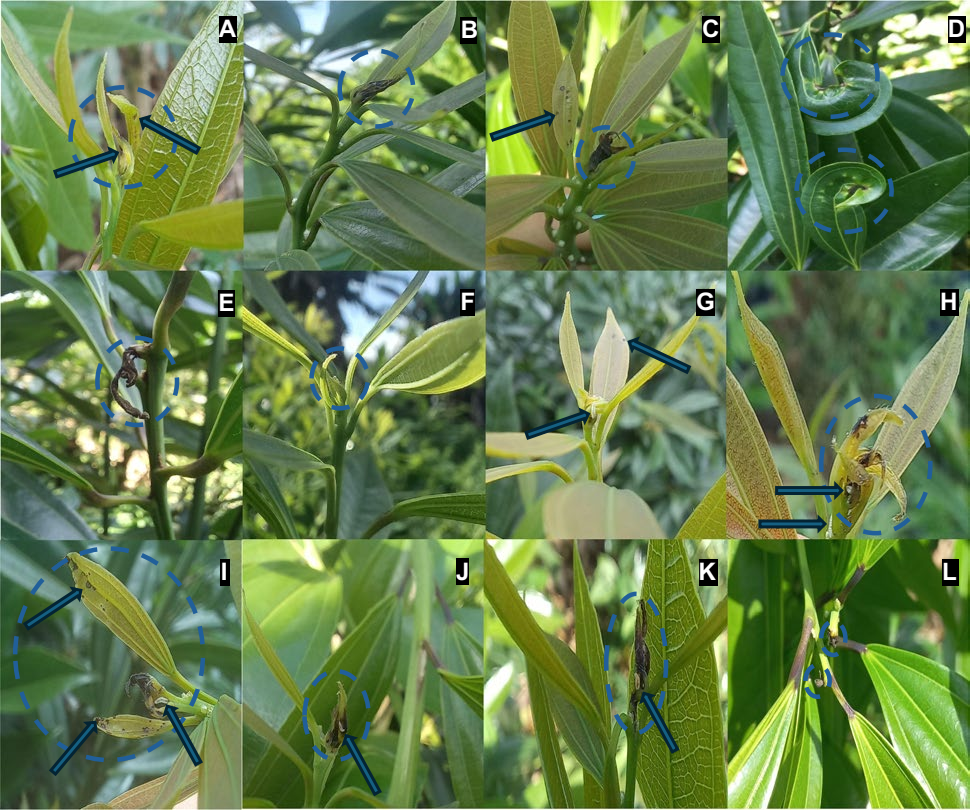
Photographic description of *Prodiplosis longifila* damage on *Cocculus laurifolius*. **A**: Shoot apex damage. **B** and **C** Leaf and stem damage. **D** and **E** Severe leaf damage. **F**–**L** Timeline of larva damage. Photographs: *Economía Agrícola*

In the case of *P. longifila* damage in *Cocculus*, this occurs in the apical part of the shoots and is characterized by causing a lesion in the tissue that starts as small circular spots that later merge and completely dry out this area of the shoot (Fig. 5A). Furthermore, they cause small circular lesions on the leaves and stem, which remain with a blackened coloring in both the juvenile stage and at the moment of maturity (Fig. 5B and C). In severe damage, deformed leaves are observed with necrotic spots accompanied by a chlorotic halo, and the apical part is completely dry (Fig. 5D) or may have been detached, which is mainly observed when the stem is mature (Fig. 5E). Figure 5 F-–L show the symptomatology from the moment the larva starts to consume the foliage until it causes total necrosis of the apex.

#### Quantification of the Economic Impact Associated With Damage Caused By Larvae to Foliage

To evaluate the economic impact of *P. longifila* on foliage crops, baseline expectations were established under optimal production conditions, assuming adequate nutrition, phytosanitary management, and favorable edaphoclimatic factors (Table 2). Under these conditions, projections from *Economía Agricola* indicated that approximately 36% of stems per square meter would qualify as quality A (premium export), 50% as quality B (secondary export), and 14% as quality C or discarded for the domestic market. Expected revenues were USD 3.52/m^2^ for *R. aculeatus* and USD 1.70/m^2^ for *C. laurifolius*, with quality A representing the highest share of income (USD 1.80/m^2^ for *R. aculeatus* and USD 1.20/m^2^ for *C. laurifolius*). Production costs per cycle, including labor and inputs, totaled USD 1263 for *R. aculeatus* (2 months) and USD 1534 for *Cocculus* (3 months).

**Table 2:** Comparison of infestation levels and economic impact of *Prodiplosis longifila* in foliage (*Ruscus aculeatus* and *Cocculus laurifolius*)

Infestation level^1^	Crop^2^	Yield reduction (%)^3^	Harvested stems (%) ^4^	Quality A revenue (USD/m^2^)^5^	Quality B revenue (USD/m^2^)	Quality C revenue (USD/m^2^)	Total revenue (USD/m^2^)	Income reduction (%)
No infestation	*Ruscus*	0.00	100	1.80	1.59	0.12	3.52	0.00
	*Cocculus*	0.00	100	1.20	1.00	0.06	1.70	0.00
High	*Ruscus*	53.33	46.67	0.20	0.10	0.40	0.72	79.54
	*Cocculus*	29.59	70.40	1.04	0.21	0.14	1.40	46.81
Medium	*Ruscus*	28.89	71.11	2.88	0.02	0.14	3.05	13.34
	*Cocculus*	15.78	84.22	1.58	0.24	0.04	1.87	17.20
Low	*Ruscus*	0.00	91.97	4.34	0.10	0.03	4.48	0.00
	*Cocculus*	0.00	88.84	2.08	0.11	0.01	2.22	2.04

^1^Infestation level: percentage of incidence (% of stems affected) defined based on the monitoring data of the current year. ^2^Crop: type of plant species evaluated. ^3^Yield reduction (%): percentage reduction in total yield due to infestation, calculated as (control yield − infested yield)/control yield × 100. ^4^Harvested Stems (%): percentage of marketable stems suitable for harvest, excluding those damaged by pests (> 20% damage). ^5^Quality A/B/C revenue (USD/m^2^): revenue per m^2^ according to commercial quality categories (A: premium without defects; B: acceptable with minor damage; C: low quality), based on local market prices (USD). ⁶Total revenue (USD/m^2^): sum of revenue for all qualities (A + B + C). Income reduction (%)**⁷: percentage reduction in total revenue due to infestation, compared to a scenario without pests: (control revenue − infested revenue)/control revenue × 100

Under high infestation, yield reductions reached 53.33% in *R. aculeatus* and 29.59% in *C. laurifolius*. Of the stems harvested (46.67% and 70.40%, respectively), none with *P. longifila* damage was classified as quality A, representing a total loss in this category. Only 2.99% of *R. aculeatus* (USD 0.11/m^2^) and 1.65% of *C. laurifolius* (USD 0.22/m^2^) were accepted as quality B, with 10–15% conditioned to meet minimum standards. The largest proportion, 40.02% of *R. aculeatus* and 42.70% of *Cocculus*, were relegated to the domestic market, generating USD 0.40 m^2−1^ and USD 0.14m^2^, respectively. Within this fraction, 80–90% of *R. aculeatus* and 60–70% of *Cocculus* stems showed pest-related damage. As a result, total revenues declined by 79.54% in *R. aculeatus* and 46.81% in *Cocculus* compared to projections (Table 2).

Under medium infestation, yield losses were 28.89% in *Ruscus* and 15.78% in *C. laurifolius*. Of the harvested stems (71.11% and 84.22%), none with visible damage reached quality A. A small fraction entered quality B: 2.67% of *R. aculeatus* (USD 0.022/m^2^) and 16.96% of *C. laurifolius* (USD 0.24/m^2^), with 10–15% conditioned stems. The domestic market absorbed 2.59% of *R. aculeatus* (USD 0.14/m^2^) and 12.53% of *C. laurifolius* (USD 0.049/m^2^), with 65–75% and 55–60% of stems, respectively, showing *P. longifila* damage. Overall, revenues decreased by 13.34% in *R. aculeatus* and 17.2% in *Cocculus*.

Under low infestation, harvest proportions remained high (91.97% in *R. aculeatus* and 88.84% in *C. laurifolius*). Export allocation was dominant, with 90.95% of *Ruscus* and 95.83% of *Cocculus* stems meeting export standards. A minor fraction entered quality B: 3.45% of *Ruscus* (USD 0.11/m^2^) and 7.8% of *Cocculus* (USD 0.12/m^2^), with 10–15% (*R. aculeatus*) and 7–10% (*C. laurifolius*) conditioned stems. The domestic market represented only 1.79% of *R. aculeatus* (USD 0.035/m^2^) and 4.15% of *Cocculus* (USD 0.017/m^2^), with 5–7% of stems damaged. Under these conditions, revenues not only remained stable but exceeded expectations in *R. aculeatus* (USD 4.34 m^2−1^ vs. 3.52 m^2−1^ projected), while *Cocculus* experienced a minimal reduction of 2.04% (Table 2).

A management plan based on phytosanitary applications was implemented to reduce *P. longifila* populations in foliage crops. This strategy imposes a substantial economic burden, as most resources are directed specifically to pest control. In *R. aculeatus* greenhouses with high and medium infestations, management practices were similar, resulting in no major cost differences. In contrast, in low-infestation greenhouses, a key distinction was the absence of continuous drench applications. For *Cocculus*, management practices were uniform across plots regardless of infestation level. In *R. aculeatus*, one production cycle required USD 1079 for crop protection in greenhouses with high and medium infestations, of which USD 780 (73.37%) corresponded to *P. longifila* management. Labor costs totaled USD 442, with COP 1,367,675 directly linked to this pest. Under low infestation, expenses declined to USD 64.70, largely due to fewer drench applications, representing only 11.8% of the total. The remaining 26.63% of costs were associated with fungal, bacterial, and arthropod control, mollusk management, and foliar fertilization. On average, application costs reached USD 0.37/m^2^ in *R. aculeatus*, with USD 0.28/m^2^ devoted to *P. longifila* (Table 3). In *C. laurifolius*, a production cycle required USD 589 for phytosanitary applications, with USD 353 (plus USD 85 in labor) directed to *P. longifila* control. Unlike *R. aculeatus*, costs did not vary by infestation level, averaging USD 0.21/m^2^, of which USD 0.14/m^2^ were associated with this pest (Table 3).

**Table 3:** Comparison of application costs at different infestation levels of *Prodiplosis longifila*

Infestation level^1^	Crop	Chemical products cost (**USD**)	Products for *Prodiplosis* (**USD**)	Labor for *Prodiplosis* (**USD**)	Total laboral cost (**USD**)	Total application cost (**USD**)	Total *Prodiplosis* cost (**USD**)	Resources for *Prodiplosis* control (%)
No infestation	*Ruscus*	299	0	0	106	405	0	0.00
	*Cocculus*	235	0	0	125	360	0	0.00
High	*Ruscus*	1079	780	336	442	1521	1116	73.37
	*Cocculus*	589	353	85	210	674	438	65.03
Medium	*Ruscus*	1079	780	336	442	1521	1116	73.37
	*Cocculus*	589	353	85	210	674	438	65.03
Low	*Ruscus*	1014	737	314	420	1434	1051	73,28
	*Cocculus*	589	353	85	210	674	438	65.03

^1^Infestation level: percentage of incidence (% of stems affected) defined based on the monitoring data of the current year. ^2^Crop: crop assessed. ^3^Chemical products cost (USD): total cost of general chemical products (insecticides, fungicides) (USD), per unit area. ^4^Products for *Prodiplosis* (USD): specific cost of chemicals targeting *Prodiplosis*, excluding other inputs. ^5^Labor for *Prodiplosis* (USD): labor cost for specific application against *Prodiplosis* (wages × daily rate, in USD). ⁶Total labor cost (USD): sum of all labor costs (general + specific applications). ⁷Total application cost (USD): total cost of applications (chemicals + labor). ⁸Total *Prodiplosis* cost (USD): total cost of *Prodiplosis* control (specific products + specific labor). ⁹Resources for *Prodiplosis* control (%): percentage of total production budget allocated to *Prodiplosis* control: (*Prodiplosis* cost/total production cost) × 100

### Experimental Design for Capturing Adults of *P. Longifila* in Foliage Crops

**Fig. 6:**
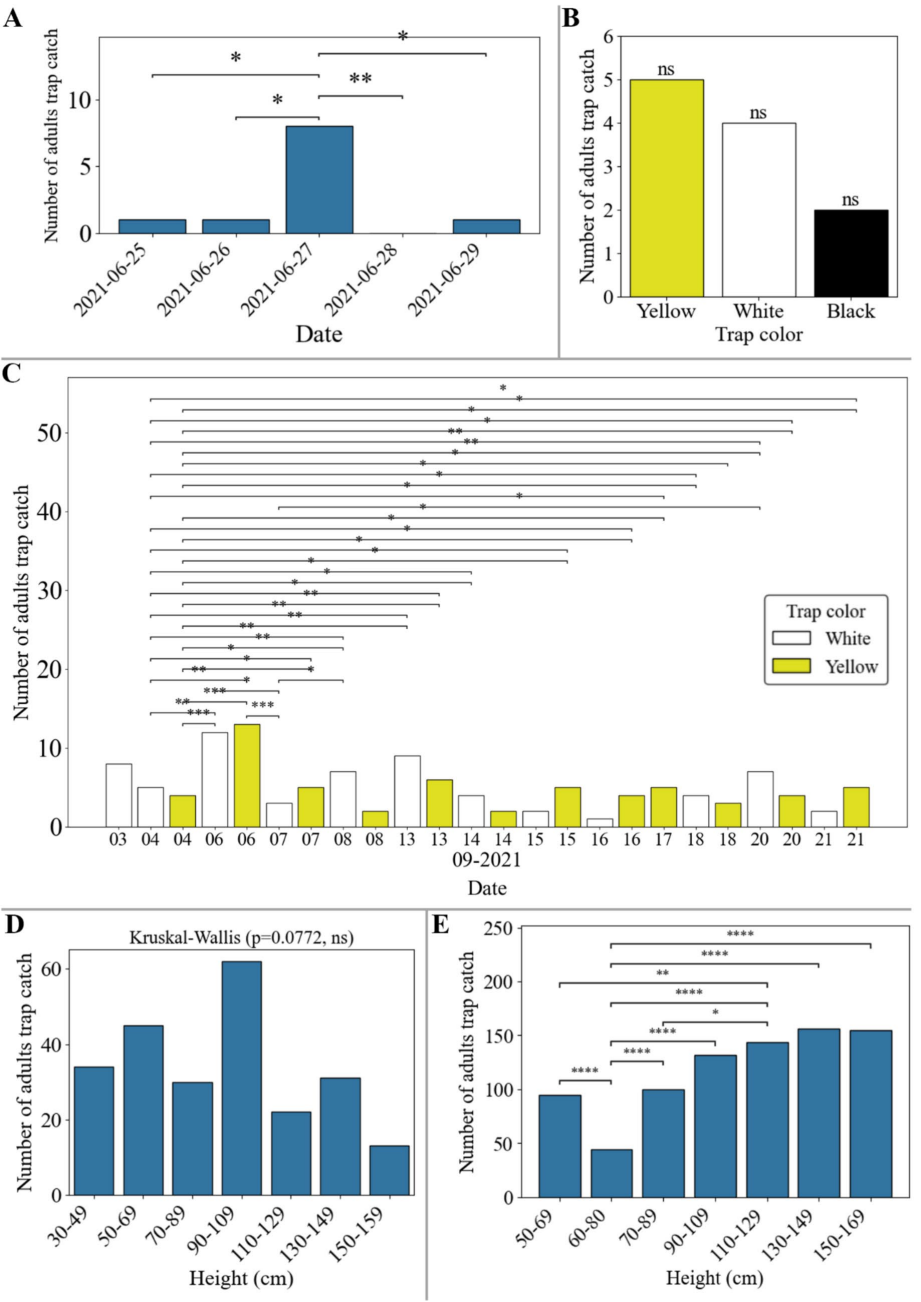
Population dynamics of *Prodiplosis longifila* in foliage associated with different trap characteristics (color and height) and measurement dates. **A** Population fluctuation in trial 1 based on measurement dates. **B** Population fluctuation in trial 1 based on trap color. **C** Population fluctuation in trial 2 based on trap color and date.** D** Population fluctuation in trial 3 based on trap height. 50–69 cm had significant differences between 30–49 cm and 150–159 cm (Mann–Whitney post hoc).** E** Population fluctuation in trial 4 based on trap height with p = 2 × 10^–7^(minimum 60–80 cm (< 50 adults); maximum 130–149 cm (> 150 adults)). Black asterisks: significant differences Mann–Whitney (consecutive pairs): ***p ≤ 0.001. **p ≤ 0.01. *p ≤ 0.05. ns *p* > 0.05

The results obtained from the trials revealed important patterns in the capture of adult *P. longifila* using chromatic traps of different colors and heights in greenhouses, identifying significant effects of trap color and height on the effectiveness of capturing this pest (Fig. 6; Supplementary Material: Fig. S2, S3). In trial 1, the results of the Kruskal–Wallis statistical test showed significant differences when comparing the number of adults by date (*p*-value 0.0024) using Mann–Whitney comparisons, where on September 27, more than five adults were trapped (Fig. 6A), but no significant differences were found for trap color (*p*-value 0.4899) (Fig. 6B).

In trial 2, monochromatic white traps captured 64 adults of *P. longifila* compared to 58 in yellow traps, for an absolute difference of 6 adults, but there were no significant differences by color (Kruskal–Wallis, *p-*value 0.852). Temporal effects (*p-*value 2.1 × 10^–8^) and date × color interaction (*p-*value 8.8 × 20^–6^) were detected, suggesting a preferential attraction of adults to the color white, which could be related to their crepuscular behavior and the light conditions of the environment. The total number of individuals captured was highest on September 6, with more than 10 adults (Fig. 6C), but on September 4 there were significant differences with the other values; considering that there were 18 values of 0 and that the test is based on distribution, there is high variability in the data, which indicates the need to increase the numbers of traps and replicates.

In trial 3, the test results showed no significant differences in the number of adults captured by color regardless of the date of evaluation and the height of the trap (*p*-value 0.8125). For height, no significant differences were obtained (*p*-value 0.0772) using the Kruskal–Wallis test, reporting only descriptive trends (Fig. 6D), whereby traps placed at heights of 150–159 cm recorded the lowest number of captures, while those placed at heights of 90–109 cm had the highest number of captures. For the date, differences were determined, with higher values of adults trapped from December 9 onwards, and both the interaction of date of assessment and the color and height of the traps have a significant effect on the capture of *P. longifila* adults (Supplementary Material: Fig. S2).

In trial 4, there were significant differences between trap heights (*p-*value 2 × 10^–7^). Specifically, the 60–80 range captured fewer than 50 adult *P. longifila* showing significant differences from the other heights (*p-*value ≤ 1 × 10^–4^, and the ranges 90–109 to 150–169 showed no significant differences, with more than 125 adults captured (Fig. 6E). In this trial, it was observed that traps located at higher heights tended to capture more adults, which could indicate that the insects are more frequently found in the upper layers of the plant canopy in greenhouses. The results of trials 3 and 4 differ in terms of the total number of adults captured, but their distribution may be similar, as intrinsic factors such as plant size or phenology at the time of assessment may influence population dynamics and adult flight behavior above the canopy surface, where they expend less energy and are then attracted by factors such as trap color.

In trial 5 in R1-SA, black and red traps had the highest average values of 4.9 and 4.8 respectively, but in terms of the total number of adults trapped, red was the highest with 24 adults (Table S2). The colors that obtained the highest number of adults at the end of the trial were black and red with 93 and 90 adults, respectively, while the colors blue and green recorded the lowest values, with 43 adults for both cases. Significant differences in the number of adults trapped were obtained for date (*p-*value 7.8 × 10^–14^), plot (*p-*value 9.4 × 10^–6^), infestation level (*p-*value 2.5 × 10^–6^), and trap color (*p-*value 0.0065), but not for host species (*p*-value 0.6533) based on the Kruskal–Wallis test. Multiple comparisons were then performed for each variable (Supplementary Material: Fig. S4).

**Fig. 7:**
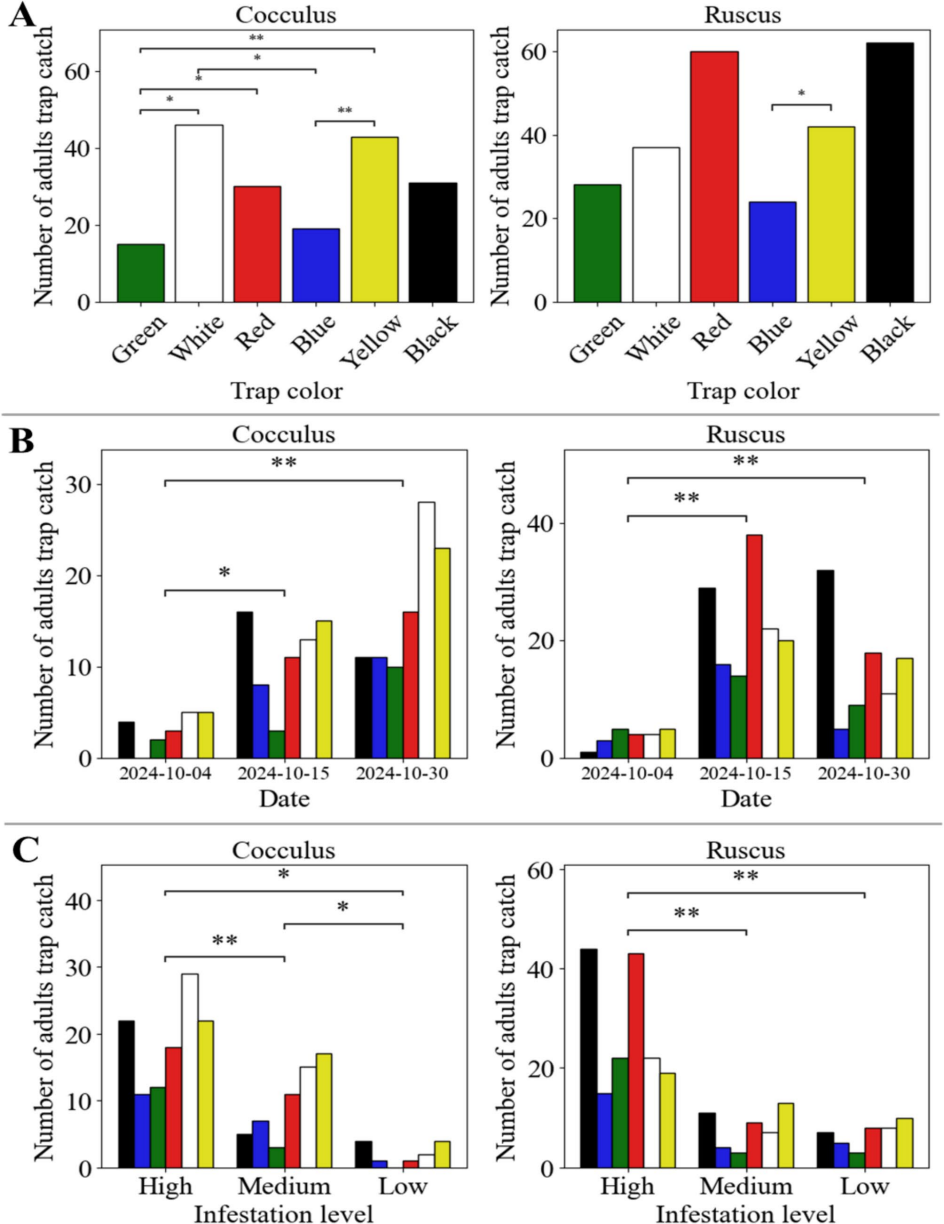
Population dynamics of *Prodiplosis longifila* in foliage for two host species. A Population fluctuation based on trap color. **B** Population fluctuation based on trap color grouped by date of assessment. **C** Population fluctuation based on trap color grouped by infestation level. In each figure, the evaluated variables that captured a total number of adults with significant differences are indicated with black asterisks depending on the *p*-value: ***p ≤ 0.001. **p ≤ 0.01. *p ≤ 0.05. ns *p* > 0.05

Further analysis was performed for all variables grouped by host species, with results showing differences in the distribution of adult trap catches by color (Supplementary Material: Fig. S4A). For *C. laurifolius*, more adults were captured with yellow and white traps, with green traps showing greater differences compared to other colors, while for *Ruscus* more adults were captured with red and black traps (Fig. 7A). Both *R. aculeatus* and *C. laurifolius* showed significant differences between blue and yellow, with lower values for blue (Fig. 7A).

Regarding the date, they show that for both *C. laurifolius* and *R. aculeatus*, the lowest number of adults was recorded on the date of the first assessment, and this had significant differences with the subsequent assessment dates regardless of the color of the traps (Supplementary Material: Fig. S4B). This was especially the case with *Cocculus*, where the highest number of adults, at around 100, was recorded on October 30, while for *Ruscus* the highest number was recorded on October 15, with a little less than 150 adults (Fig. 7B).

With regard to infestation level, it is observed that both *C. laurifolius* and *R. aculeatus* have greater numbers of adults trapped with high infestation level (Supplementary Material: Fig. S4C), but there are no significant differences between high and medium infestation levels for *C. laurifolius*, while for *R. aculeatus* there are no significant differences for medium and low infestation levels (Fig. 7C).

**Fig. 8:**
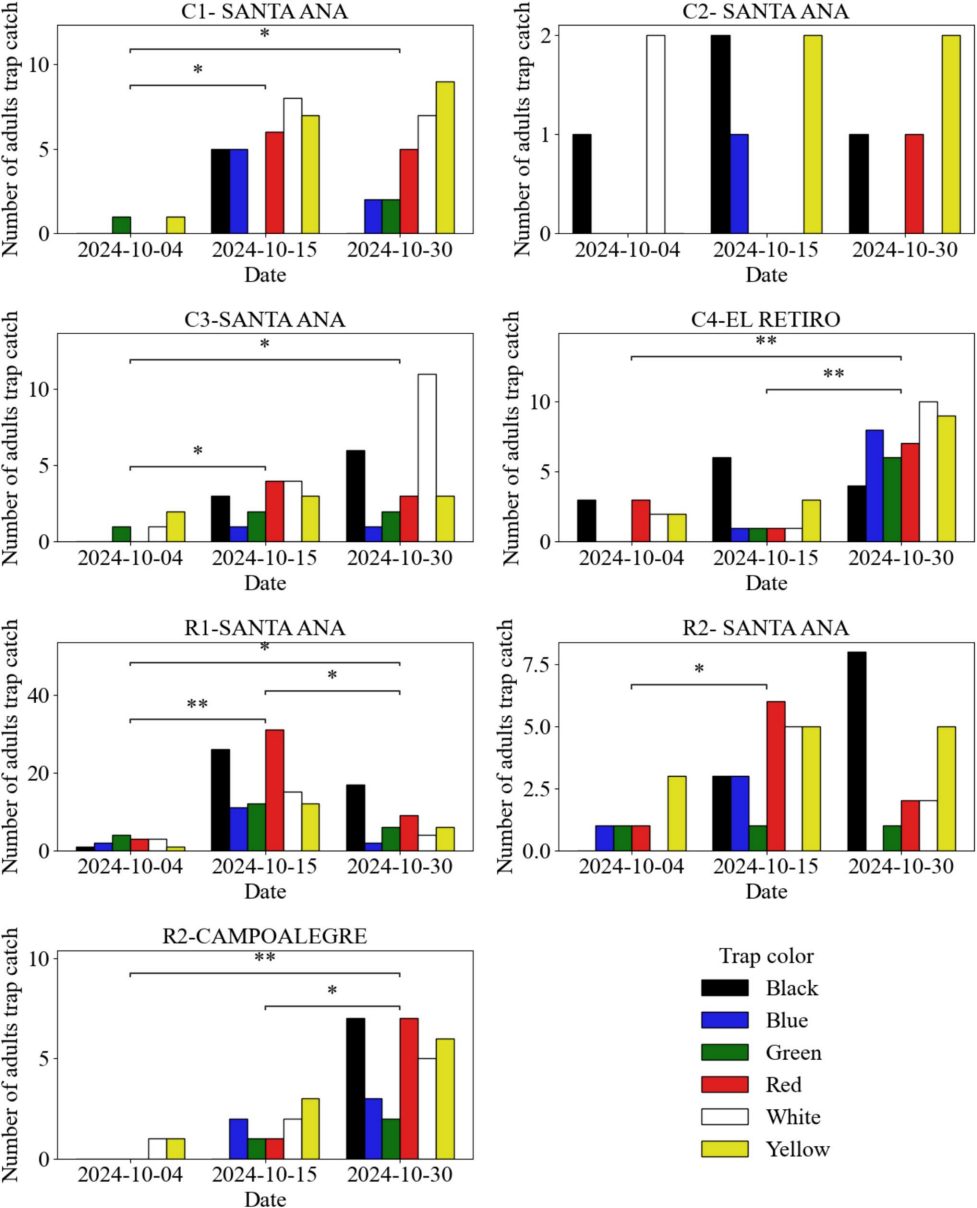
Population dynamics of *Prodiplosis longifila* in foliage by plot and date. Colored bars represent total adult captures by trap color. Black asterisks (*) indicate significant differences between consecutive dates (Mann–Whitney post hoc test, *p* < 0.05) within each plot ***p ≤ 0.001. **p ≤ 0.01. *p ≤ 0.05. ns *p* > 0.05)

At the farm and plot level for *R. aculeatus*, Santa Ana R1 had the highest number of adults trapped, with more than 100 individuals, mainly in red and black traps, on October 15 (Fig. 8), showing significant differences from the other plots evaluated (Supplementary Material: Fig. S4C). Meanwhile, Santa Ana R2 and Campo Alegre R2 did not show significant differences between each other. In the case of *C. laurifolius*, Santa Ana C2 had the lowest number of adults trapped, with less than two individuals, predominantly by black and yellow traps (Fig. 8), with no significant differences between the plots of the Santa Ana and El Retiro farms. These results show that although no significant differences were identified between host plants in terms of the number of adults trapped, the differences between plots show not only that the pest is distributed throughout all locations within the farms evaluated, but also that it may have a higher degree of concentration at the time of evaluation, as a result of control measures, environmental conditions within the plots, or historical incidence of the pest.

### Temporal and Spatial Dynamics of Adult and Immature Populations and Damage Associated With *P. Longifila* in Foliage

**Fig. 9:**
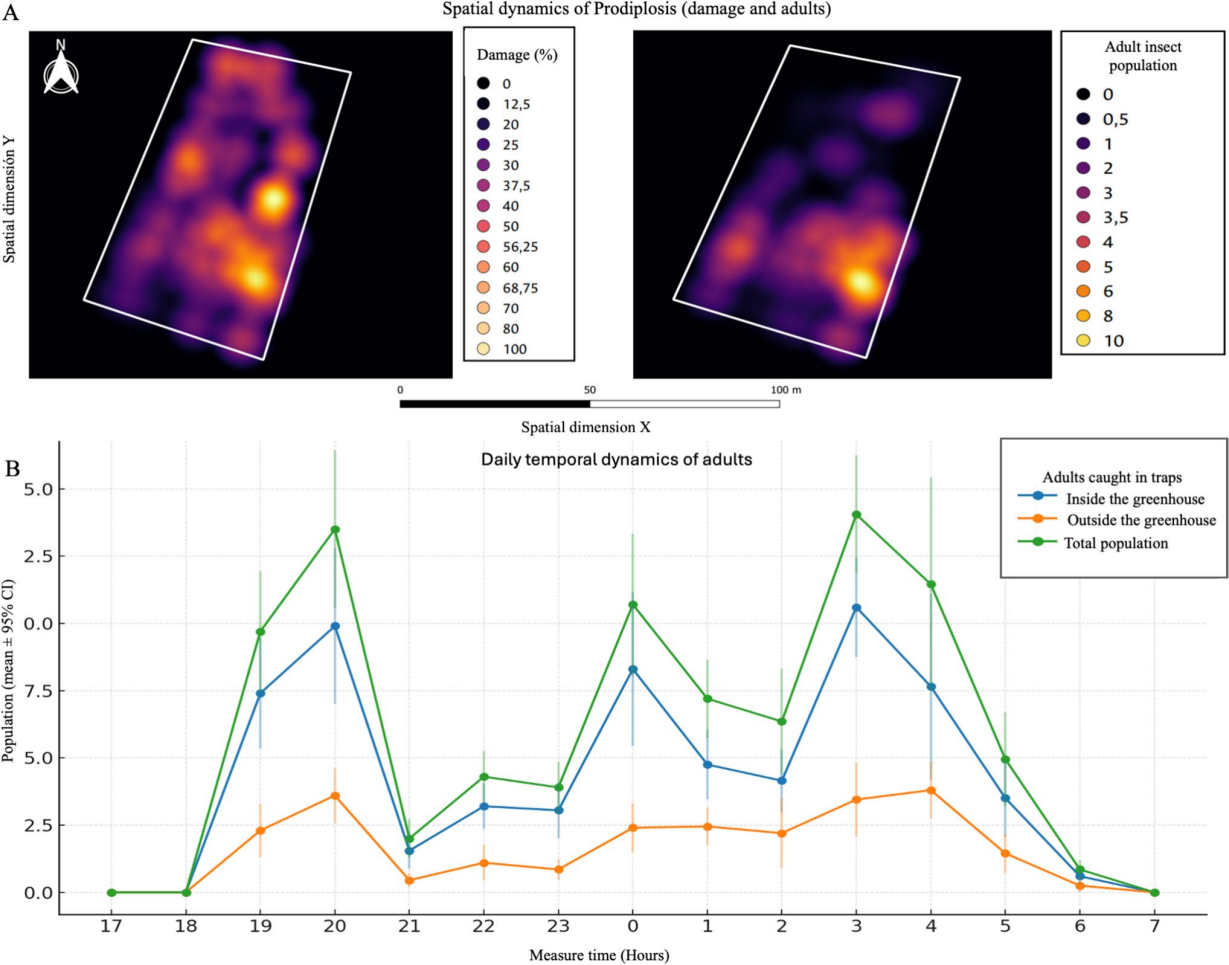
Spatio-temporal dynamics of *Prodiplosis longifila* populations: **A** Kernel density estimation (KDE) illustrating the spatial distribution and intensity of *Prodiplosis* population and damage across the study area. **B** Temporal dynamics of adult *Prodiplosis* captured hourly in traps placed inside and outside greenhouses cultivated with *Ruscus aculeatus*, showing daily activity patterns

The spatio-temporal dynamics of *P. longifila* populations and their associated damage on the host *R. aculeatus* were analyzed through the application of kernel density estimation models. This methodology enabled the delineation of non-random spatial structures within the greenhouse environment, offering a more refined understanding of pest distribution. The results consistently indicated that *P. longifila* exhibited a highly aggregated distribution, with infestation foci concentrated along structural edges, proximal to entry points, and in low-light microhabitats within the greenhouse. Such distributional patterns underscore the interaction between insect behavioral ecology and greenhouse architecture, which jointly influence pest colonization and persistence (Fig. 9A).

Complementarily, the temporal component of the study was evaluated by continuous monitoring of adult captures using yellow sticky traps strategically placed both inside and outside the greenhouses over a 24-h cycle. This fine-scale temporal analysis revealed a bimodal activity pattern (Fig. 9B): an initial peak at dusk, between 19:00 and 20:00 h, followed by a more pronounced peak between 03:00 and 04:00 h. These findings suggest intrinsic behavioral rhythms linked to nocturnal activity, which may be modulated by circadian regulation or microclimatic conditions. Importantly, the temporal heterogeneity observed provides critical insights for optimizing monitoring protocols and timing of control interventions.

Furthermore, comparative analyses between internal and external trap captures demonstrated that adult populations were consistently higher within greenhouses containing *Ruscus*, reinforcing the role of host plant presence and favorable microclimatic conditions in sustaining pest populations. Nonetheless, the detection of adults outside the greenhouse’s points to potential spillover into surrounding vegetation or migratory influx from alternative hosts (Fig. 8B). This evidence highlights the necessity of adopting an integrated perspective that considers both greenhouse and external agroecosystem dynamics when designing management strategies.

## Discussion

This approach provides a detailed description of the life cycle, injury, and damage caused by *P. longifila* on two economically important foliage species, *R. aculeatus* and *C. laurifolius*. These findings confirm that damage occurs across all three larval instars, but the most destructive stages are the first and second (Gagné 1986; Hernandez et al. 2015), whereas the insect’s piercing-sucking mouthparts (stiletto-shaped) penetrate epidermal tissues, injuring subepidermal parenchyma cells and injecting toxins that result in tissue necrosis and characteristic burning symptoms (Goldsmith et al. 2013; Hernandez et al. 2015).

Our results clearly demonstrate the severe impact of *P. longifila* on foliage crops, with productivity losses of up to 53% in *R. aculeatus* and 29% in *C. laurifolius*, a marked reduction in extra-quality stems, and maximum income declines of 79% and 56%, respectively. Comparable findings have been reported in other hosts, including tomato and asparagus (Pena et al. 1987; Kroschel et al. 2012; Cedano and Cubas 2012; Geraud-Pouey et al. 2022). The economic importance of this pest derives from both direct yield losses through stem mortality and deformation of new shoots and indirect losses due to quality downgrading, which shifts stems from export to domestic markets. This situation is further aggravated by the high costs of chemical control, which may involve up to sixteen insecticide applications per cycle in *R. aculeatus* and twelve in *C. laurifolius*, with substantial environmental repercussions. The absence of a comprehensive integrated pest management (IPM) strategy has intensified reliance on chemical inputs (Valbuena-Gaona et al. 2025), resulting in recurrent problems such as inappropriate dosing, lack of rotation among active ingredients, reduced pest sensitivity, and negative impacts on natural enemies.

It is important to recognize that the economic impact of *P. longifila* on foliage crops, as presented in this study, is highly influenced by market dynamics and recent changes in commercialization standards. In the second half of 2025, updated quality requirements modified the classification of stems with pest damage: while minor injuries were previously accepted as quality B after cosmetic trimming, new regulations mandate their reclassification to quality C, further reducing production value. At the same time, revenue projections and management cost estimates should not be regarded as stable over the long term, given the inherent volatility of the foliage market. Similarly to the cut flower sector, prices are driven by global supply and demand, with sharp increases during peak seasons such as Valentine’s Day and Mother’s Day, followed by declines in off-peak periods. This cyclical variability is compounded by external pressures, including exchange rate fluctuations, escalating transportation and logistics costs to maintain the cold chain, and strong international competition (Avendaño Ruiz et al. 2023; Benavides-Parra and Beltran-Pardo 2024). Collectively, these factors generate a dynamic and uncertain commercial environment in which procurement prices for suppliers fluctuate continuously.

An important finding of this study concerns the monitoring process in foliage production systems, specifically the optimization of trap parameters such as color, size, and height. Refining and standardizing methods for quantifying pest populations is essential for effective sampling and monitoring, which in turn underpins the development of evidence-based management strategies (Hutchins 1993; Binns et al. 2000). These results not only confirmed the preference of *P. longifila* adults for particular trap colors (black, white, and yellow), but also identified the optimal capture height, which was predominantly between 10 and 30 cm above the host plant. These findings are critical for improving trapping protocols, thereby enhancing the accuracy of population estimates. Optimizing these methods will facilitate early monitoring of *P. longifila*, allowing integrated pest management (IPM) intervention before economic damage occurs, contributing to more efficient and sustainable pest management, while also providing practical tools for growers to implement in the field.

Across color-based trials (experiments 1–5), *P. longifila* adults showed a consistent numerical tendency toward white, red, and black traps, although captures did not differ statistically from those recorded on yellow traps. This pattern suggests sensitivity to a broad spectral range between approximately 400 and 700 nm, a hypothesis that warrants validation with increased statistical power. Comparable responses have been reported in *Citrus* × *aurantifolia* orchards in Florida, where white traps were slightly more attractive than yellow, while red and orange traps yielded the highest captures (Pena and Duncan 1992). Regarding vertical flight activity (experiments 3 and 4), adult captures were concentrated near the host canopy, indicating limited vertical dispersal during host-associated activity. This behavior is consistent with observations in tomato crops, where adults can disperse vertically up to 5 m but primarily remain active between 1.0 and 1.7 m (Vélez Salazar 1998), and with citrus systems, where preferred flight heights average approximately 1.5 m (Pena and Duncan 1992).

In terms of dispersal dynamics, the observations revealed a classic pattern where *P. longifila* populations initially concentrate along greenhouse borders but progressively expand inward, affecting an increasing number of plants over time and establishing a criterion for priority perimeter monitoring in IPM for these foliages in the context of production in the study area. The rate of this dispersal appears to be influenced by factors such as physical barriers, canopy structure, and the application of biological or chemical control measures. These findings underscore the importance of continuous monitoring and preventive interventions at entry points and peripheral zones, where timely actions can substantially reduce pest spread and mitigate its overall impact on *Ruscus* and *C. laurifolius* production systems.

The aggregated distribution of *P. longifila* towards greenhouse edges is consistent with previous reports describing its non-uniform distribution (Pena and Duncan 1992). This clustering behavior suggests that margins provide favorable conditions for establishment or that infestations may originate from external environments, with adults migrating into the greenhouse interior. Identifying potential alternative hosts outside the production system is therefore essential for understanding broader population dynamics and for designing effective integrated pest management strategies. In addition to environmental factors, chemical communication may also contribute to aggregation processes in *P. longifila*, potentially mediating conspecific attraction during feeding or host exploitation. Although this mechanism was not evaluated in the present study, evidence from other insect systems indicates that aggregation pheromones can strongly influence spatial distribution and collective behavior, and are actively being explored for pest monitoring and management associated with crop systems (Kirk 2017). For example, studies in citrus have indicated that adult *Prodiplosis* respond to host-related cues and exhibit clustered spatial distributions that may involve chemical signaling (Pena & Duncan 1992). Further research is needed to clarify the role of pheromones or other semiochemicals in *P. longifila* and to evaluate their potential application in monitoring and management strategies.

The presence of hotspots within greenhouses further indicates that localized environmental and microclimatic conditions such as fluctuations in temperature, humidity, or light intensity facilitate the initial establishment and aggregation of *P. longifila* (Hernandez et al. 2015; Geraud-Pouey et al. 2022). Proximity to external infestation sources likely reinforces this phenomenon. Recognizing these spatial patterns is crucial for prioritizing management efforts, particularly targeted surveillance and control along greenhouse edges, to restrict further spread into the crop interior. Moreover, analyzing these patterns provides insights into dispersal capacity, aggregation tendencies, and distribution foci in relation to crop phenology, feeding and reproductive preferences (Park et al. 2024), while also helping to evaluate the influence of climate variability and management practices on population fluctuations and life cycle changes (Huang et al. 2022).

This study represents an initial contribution to the understanding of *P. longifila* in foliage production systems by characterizing in part the phenological stages important for monitoring (larvae, pupae, and adults) and population ecology under commercial conditions and developing practical tools for adult monitoring and impact assessment. In addition, the integration of trap-based adult captures with estimates of infestation levels, economic losses, and effects on productivity and quality provides a data-driven foundation for defining action thresholds and supporting evidence-based decision-making. Nevertheless, further research is required to fully elucidate the species’ life cycle under controlled and field conditions and to advance knowledge of its population ecology, including alternative hosts, migration dynamics, life tables, and interactions with natural enemies and potential biological control agents. Future multidisciplinary approaches combining ecological studies with technological tools such as automated population quantification using artificial intelligence will be essential for strengthening integrated pest management programs, reducing chemical dependence, and mitigating the economic and environmental impacts of this emerging pest in foliage crops.

## Supplementary Information

Below is the link to the electronic supplementary material.

ESM 1 (DOCX 5.17 MB)

## Data Availability

In order to guarantee the transparency, reproducibility, and replicability of our work, we have shared the data, codes, and other information to support our support from our work. Users may consult and make use of the information and analysis scripts in the following repository, which we suggest be cited if the information, part of it, or the codes are used**:** The only exception is the section on costs and economic impact, as this information is considered sensitive for the company *Economía Agraria*.
