# Supplementary material for: Evidence-Based Monitoring of Prodiplosis longifila in Foliage Crops: Damage, Economic Impact, Trapping Tools, and Population Dynamics
Source: Neotrop Entomol. 2026 Apr 15;55(1):31. doi: 10.1007/s13744-026-01379-6 (PMC13083455; doi:10.1007/s13744-026-01379-6)
Supplement: Supplementary file 1 — (DOCX 5.17 MB) [file 13744_2026_1379_MOESM1_ESM.docx]

**Supplementary Information**

**
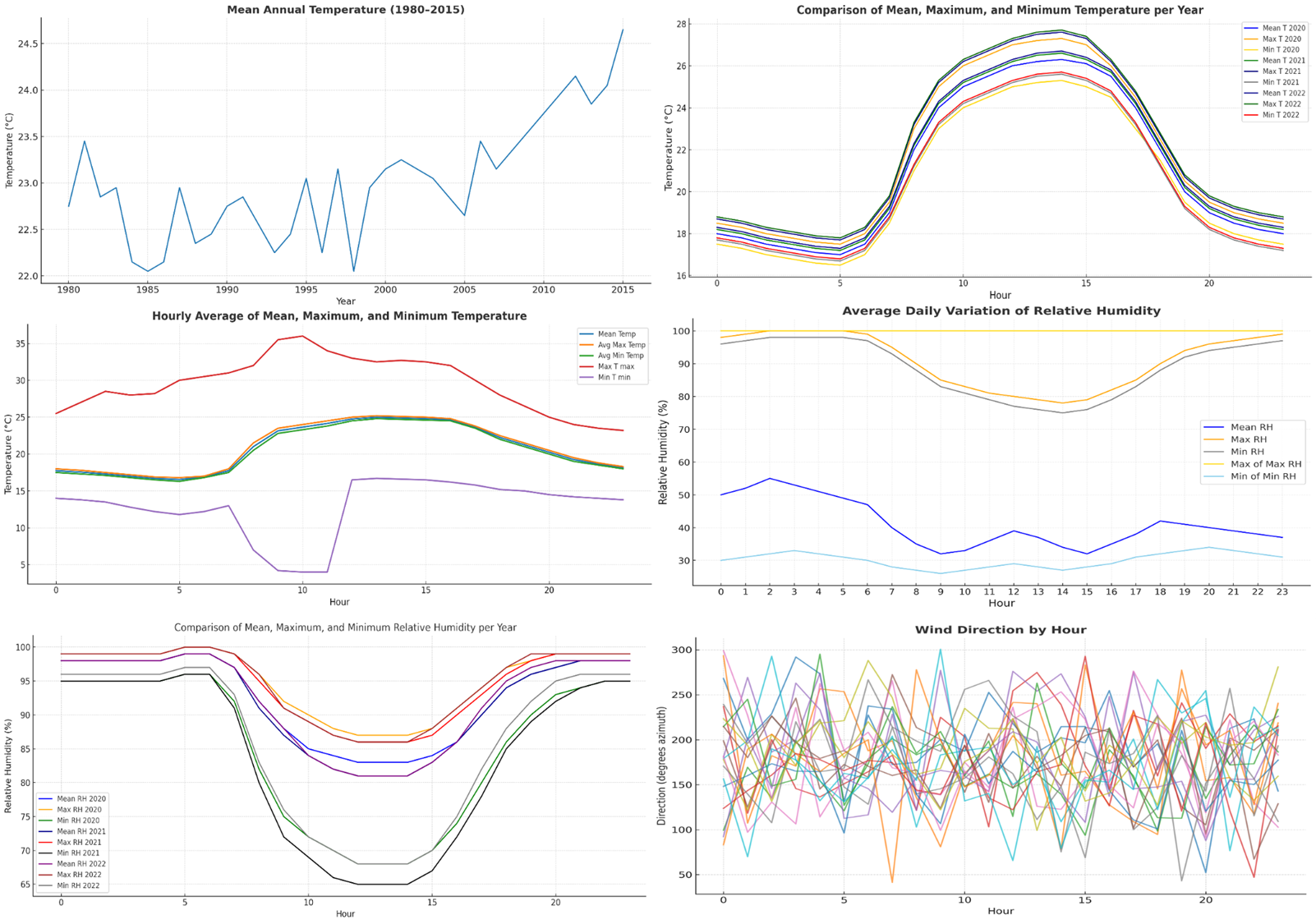
**

**Fig. S1.** Climate characterization of the study area based on synthetic data (historical series) and temperature and relative humidity sensors in greenhouses.

**
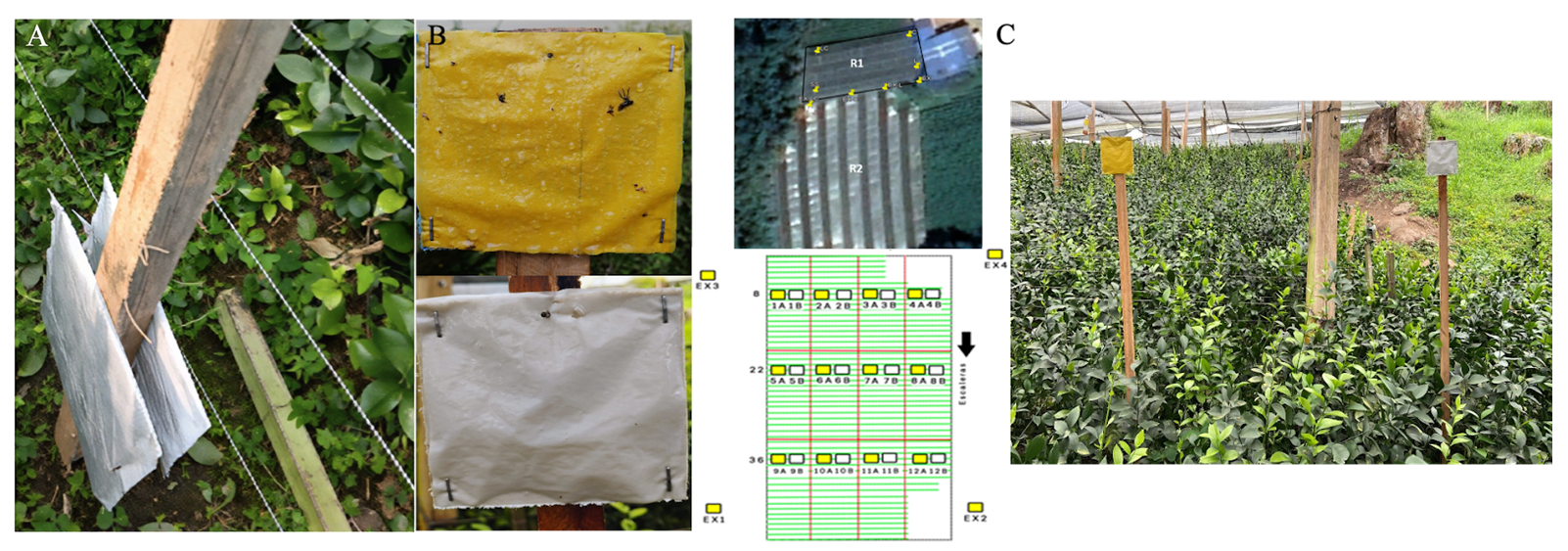
**

**Fig. S2.** Evaluation of the effect of the height and trap color on capturing adults of *Prodiplosis longifila*. **A:** Traps installed in the first trial. B**:** Yellow and white traps for all trials. C**:** Satellite view of the field, map and location of the traps for spatial dynamics analysis.

**Table S1.** Commercial quality categories for *Prodiplosis longifila* affected stems based on foliage, inflorescence, and trimming criteria.

| Quality | A | B | C (National) |
| --- | --- | --- | --- |
| Stem | Length ranging from a minimum of 55 cm to 70 cm, basal diameter of approximately 0.5 cm in *Ruscus* and 0.8 cm in Cocculus, with no twists exceeding a 2 cm deviation from the central axis. | Minimum length of 40 cm, with no twists exceeding a 3 cm deviation from the central axis. | Stem length greater than 45 cm; stem curvature is accepted, and stem diameter is not considered a limiting factor. |
| Foliage | Continuous foliar arrangement along the first 30–35 cm, with 35 cm considered the optimal value. The apical region presents an erect leaf (Cocculus) or phyllode (*Ruscus*). Apical leaf burn is less than 1 mm; when ranging between 1 and 2 mm, postharvest aesthetic conditioning is required. | Continuous foliar arrangement along the first 30 cm, the apical region may present an erect or slightly flattened leaf (Cocculus) or phyllode (*Ruscus*). Minor malformations are permitted. Apical leaf burn is less than 2 mm, above this threshold, postharvest aesthetic conditioning is required. A slightly lime-green tone is tolerated. | No specific length is defined for foliar (Cocculus) or phyllode (*Ruscus*) arrangement along the stem. Malformations are permitted. Apical leaf burn is less than 3 mm, and no postharvest aesthetic conditioning is performed. Coloration is darker green and may exhibit chlorotic mottling or textural alterations of the foliage, such as stippling caused by feeding damage by *P. longifila*. |
| Inflorescence | Absence of inflorescence. | Absence of inflorescence. | Inflorescence may be present in *Ruscus.* |

**Table S2.** Total catches of *Prodiplosis longifila* individuals in six trap colors in seven plots coded as *Cocculus* *laurifolius* (C) or *Ruscus aculeatu*s (R), and Santa Ana (SA), El Retiro (ER), and Campo Alegre (CA) farms with time series of four samples in trial 5. M is the sample mean, SD is the standard deviation.

|  | M | SD | Min | Max |  | M | SD | Min | Max |  | M | SD | Min | Max |
| --- | --- | --- | --- | --- | --- | --- | --- | --- | --- | --- | --- | --- | --- | --- |
| Plot | *Black* | | | |  | *Blue* | | | |  | *Green* | | | |
| C1-SA | 0.8 | 2 | 0 | 5 |  | 1.2 | 1.2 | 0 | 3 |  | 0.5 | 0.8 | 0 | 2 |
| C2-SA | 1.3 | 0.6 | 1 | 2 |  | 0.3 | 0.6 | 0 | 1 |  | 0 | 0 | 0 | 0 |
| C3-SA | 1 | 1.2 | 0 | 4 |  | 0.2 | 0.4 | 0 | 1 |  | 0.6 | 0.5 | 0 | 1 |
| C4-ER | 1.4 | 1 | 0 | 3 |  | 1 | 1.3 | 0 | 3 |  | 0.8 | 1.1 | 0 | 3 |
| R1-SA | 4.9 | 4.3 | 0 | 11 |  | 1.7 | 2 | 0 | 6 |  | 2.4 | 1.6 | 1 | 6 |
| R2-SA | 1.2 | 2.1 | 0 | 6 |  | 0.4 | 0.5 | 0 | 1 |  | 0.3 | 0.5 | 0 | 1 |
| R2-CA | 0.8 | 1.7 | 0 | 5 |  | 0.6 | 0.7 | 0 | 2 |  | 0.3 | 0.7 | 0 | 2 |
|  | *Red* | | | |  | *White* | | | |  | *Yellow* | | | |
| C1-SA | 1.8 | 1.6 | 0 | 4 |  | 2.5 | 2.3 | 0 | 5 |  | 2.8 | 2.3 | 0 | 6 |
| C2-SA | 0.3 | 0.6 | 0 | 1 |  | 0.7 | 1.2 | 0 | 2 |  | 1.3 | 1.2 | 0 | 2 |
| C3-SA | 0.8 | 0.7 | 0 | 2 |  | 1.8 | 1.9 | 0 | 5 |  | 0.9 | 0.6 | 0 | 2 |
| C4-ER | 1.2 | 1.1 | 0 | 3 |  | 1.4 | 1.9 | 0 | 6 |  | 1.6 | 1.3 | 0 | 4 |
| R1-SA | 4.8 | 7.5 | 0 | 24 |  | 2.4 | 2.7 | 0 | 8 |  | 2.1 | 2.6 | 0 | 8 |
| R2-SA | 1 | 1 | 0 | 2 |  | 0.8 | 1.3 | 0 | 4 |  | 1.4 | 1.1 | 0 | 4 |
| R2-CA | 0.9 | 1.6 | 0 | 5 |  | 0.9 | 0.8 | 0 | 2 |  | 1.1 | 0.9 | 0 | 2 |


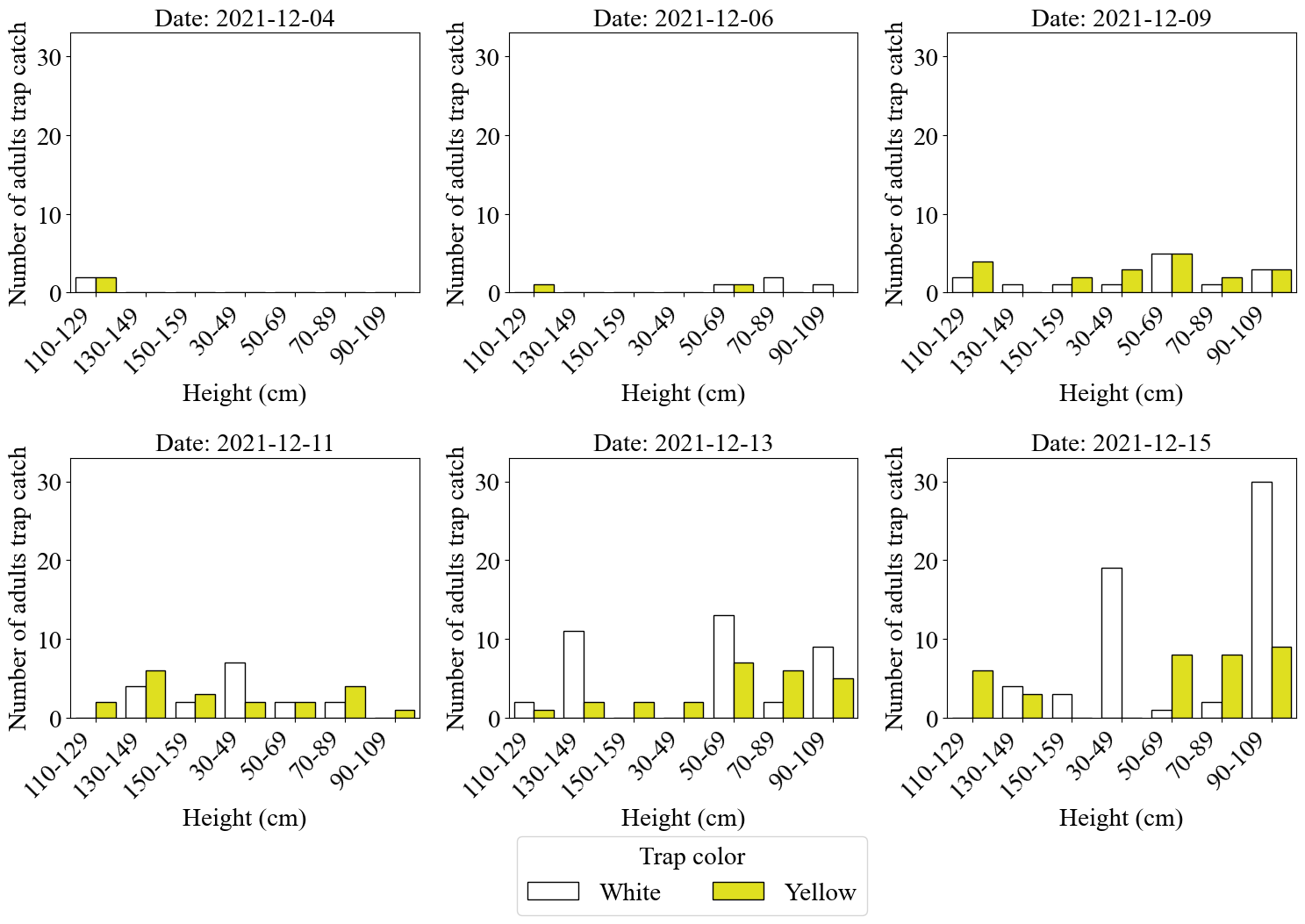


**Fig. S3.** Evaluation of the effect of the date, height and trap color on capturing adults of *Prodiplosis longifila* for trial 3.

**
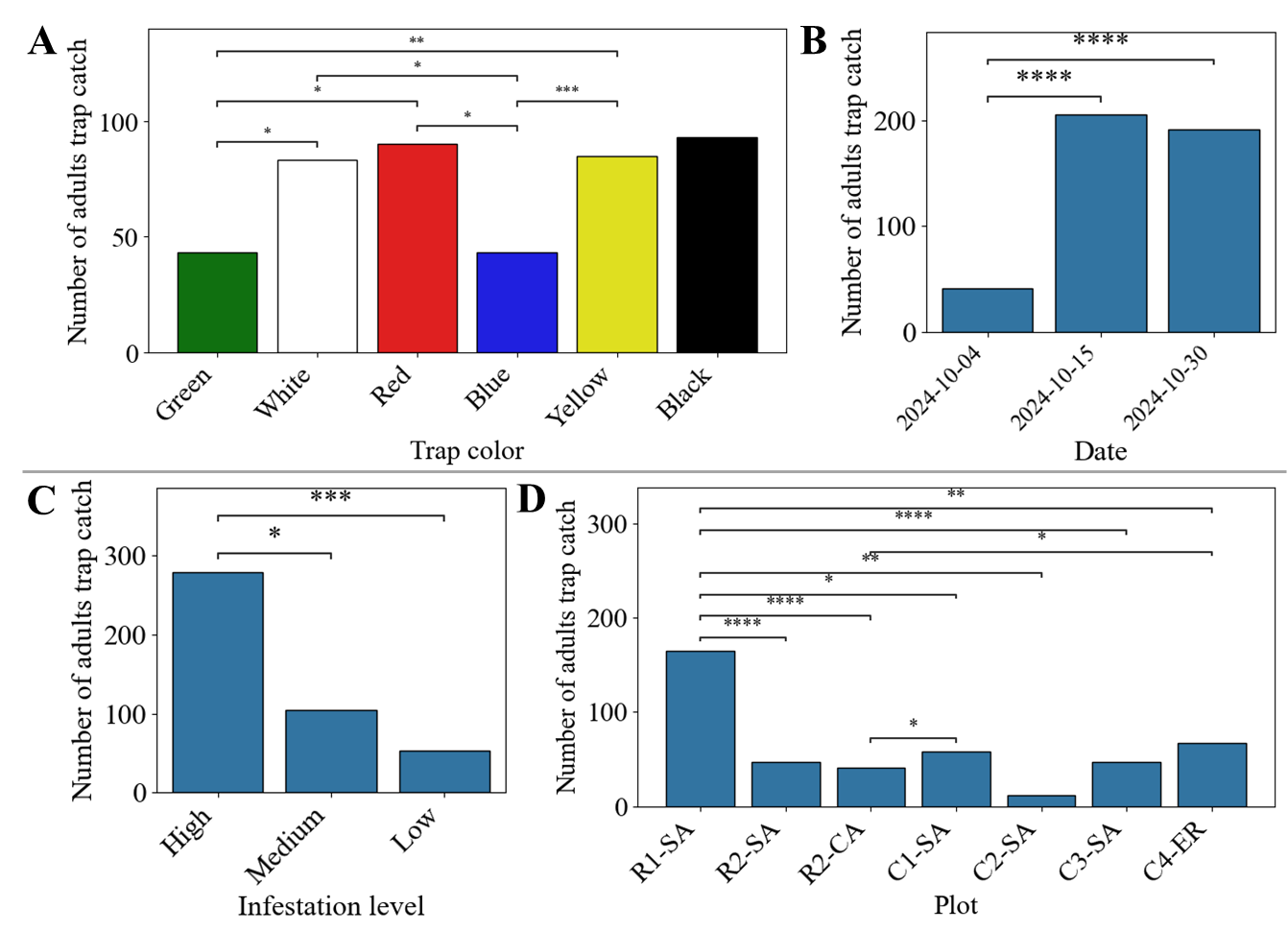
**

**Fig. S4.** Population dynamics of *Prodiplosis longifila* in foliage. **A.** Population fluctuation based on trap color; **B.** Population fluctuation based on date of assessment; **C.** Population fluctuation based on infestation level; **C.** Population fluctuation based on plot. In each figure, the evaluated variables that captured a total number of adults with significant differences are indicated with black asterisks depending on the p-value: * = 0.01 < p ≤ 0.05; ** = 0.001 < p ≤ 0.01; *** = 0.0001 < p ≤ 0.001; **** = p ≤ 0.0001.
